# Tumor loss-of-function mutations in *STK11/LKB1* induce cachexia

**DOI:** 10.1172/jci.insight.165419

**Published:** 2023-04-24

**Authors:** Puneeth Iyengar, Aakash Y. Gandhi, Jorge Granados, Tong Guo, Arun Gupta, Jinhai Yu, Ernesto M. Llano, Faya Zhang, Ang Gao, Asha Kandathil, Dorothy Williams, Boning Gao, Luc Girard, Venkat S. Malladi, John M. Shelton, Bret M. Evers, Raquibul Hannan, Chul Ahn, John D. Minna, Rodney E. Infante

**Affiliations:** 1 Center for Human Nutrition,; 2 Department of Radiation Oncology,; 3 Harold C. Simmons Comprehensive Cancer Center,; 4 Department of Internal Medicine,; 5 Department of Population and Data Sciences,; 6 Department of Radiology,; 7 Department of Pharmacology,; 8 Hamon Center for Therapeutic Oncology Research,; 9 Department of Bioinformatics,; 10 Department of Pathology, and; 11 Department of Molecular Genetics, University of Texas Southwestern Medical Center, Dallas, Texas, USA.

**Keywords:** Metabolism, Oncology, Genetic variation, Lung cancer, Molecular genetics

## Abstract

Cancer cachexia (CC), a wasting syndrome of muscle and adipose tissue resulting in weight loss, is observed in 50% of patients with solid tumors. Management of CC is limited by the absence of biomarkers and knowledge of molecules that drive its phenotype. To identify such molecules, we injected 54 human non–small cell lung cancer (NSCLC) lines into immunodeficient mice, 17 of which produced an unambiguous phenotype of cachexia or non-cachexia. Whole-exome sequencing revealed that 8 of 10 cachexia lines, but none of the non-cachexia lines, possessed mutations in serine/threonine kinase 11 (*STK11/LKB1*), a regulator of nutrient sensor AMPK. Silencing of *STK11/LKB1* in human NSCLC and murine colorectal carcinoma lines conferred a cachexia phenotype after cell transplantation into immunodeficient (human NSCLC) and immunocompetent (murine colorectal carcinoma) models. This host wasting was associated with an alteration in the immune cell repertoire of the tumor microenvironments that led to increases in local mRNA expression and serum levels of CC-associated cytokines. Mutational analysis of circulating tumor DNA from patients with NSCLC identified 89% concordance between *STK11/LKB1* mutations and weight loss at cancer diagnosis. The current data provide evidence that tumor *STK11/LKB1* loss of function is a driver of CC, simultaneously serving as a genetic biomarker for this wasting syndrome.

## Introduction

Cancer cachexia (CC) is a wasting syndrome characterized by muscle and adipose loss that subsequently leads to weight decrement. It is observed in 50% of all patients with solid tumors, responsible for 30% of all cancer-related deaths, and associated with reduced cancer survival and quality of life (1, 2). Reversing or suppressing CC is anticipated to improve survival independent of tumor-directed therapy based on preclinical models (3, 4).

Despite an understanding of CC’s devastating effects on cancer patient quality of life and survival, more than 140 cachexia clinical studies outlined in ClinicalTrials.gov have been unable to proffer an FDA-approved therapeutic intervention for CC. This failure is in part due to the lack of CC biomarkers that would 1) allow for the enrollment of patients with early-stage cachexia into clinical trials, optimizing the chances that therapies will suppress additional wasting, and 2) allow for the elucidation of the molecular drivers of CC development. Barriers in identifying novel CC mechanisms include a relatively limited effort to study CC from a systems biology perspective that would potentially illuminate common pathways for therapeutic targeting across multiple host tissues rather than emphasizing treatments that antagonize one molecule or host tissue at a time. Furthermore, with in vitro and in vivo preclinical studies, the CC field has not had the ideal isogenic and pathology-matched control tumor lines that could facilitate a better understanding of how cachexia-inducing tumors/lines differentially promote host tissue wasting.

The evolving landscape of increased genomic testing of human tumors and derived cell lines has further given us an opportunity to leverage these findings to identify a genetic biomarker that regulates CC development through a novel in vivo human non–small cell lung cancer (NSCLC) cachexia screen. Fifty-four NSCLC lines were evaluated for their capacity to induce CC-associated adipose and muscle wasting in vivo. Whole-exome sequencing of the NSCLC lines revealed a significant concordance between an NSCLC’s capacity to induce cachexia in vivo and the presence of tumor serine/threonine kinase 11 (*STK11/LKB1*) loss-of-function variants. Creation of isogenic pairs of cancer lines and their controls demonstrated that elimination of *STK11*/*LKB1* expression in 2 primary cancers, human NSCLC and mouse colorectal cancer (CRC), conferred cachexia phenotypes when transplanted into immunocompromised and immunocompetent hosts, respectively. These *STK11*/*LKB1* mutant tumors demonstrated cellular changes in their microenvironments conducive for increasing systemic pro-inflammatory cytokine levels associated with host wasting. Circulating tumor DNA analysis of a cohort of 246 NSCLC patients revealed that 90% of patients with an *STK11*/*LKB1* variant demonstrated weight loss at cancer diagnosis. Overall, our studies highlight the role of *STK11*/*LKB1* in regulating CC development and as a potential tumor-specific CC biomarker.

## Results

### Cachexia screen of human NSCLCs.

**Figure 1:**
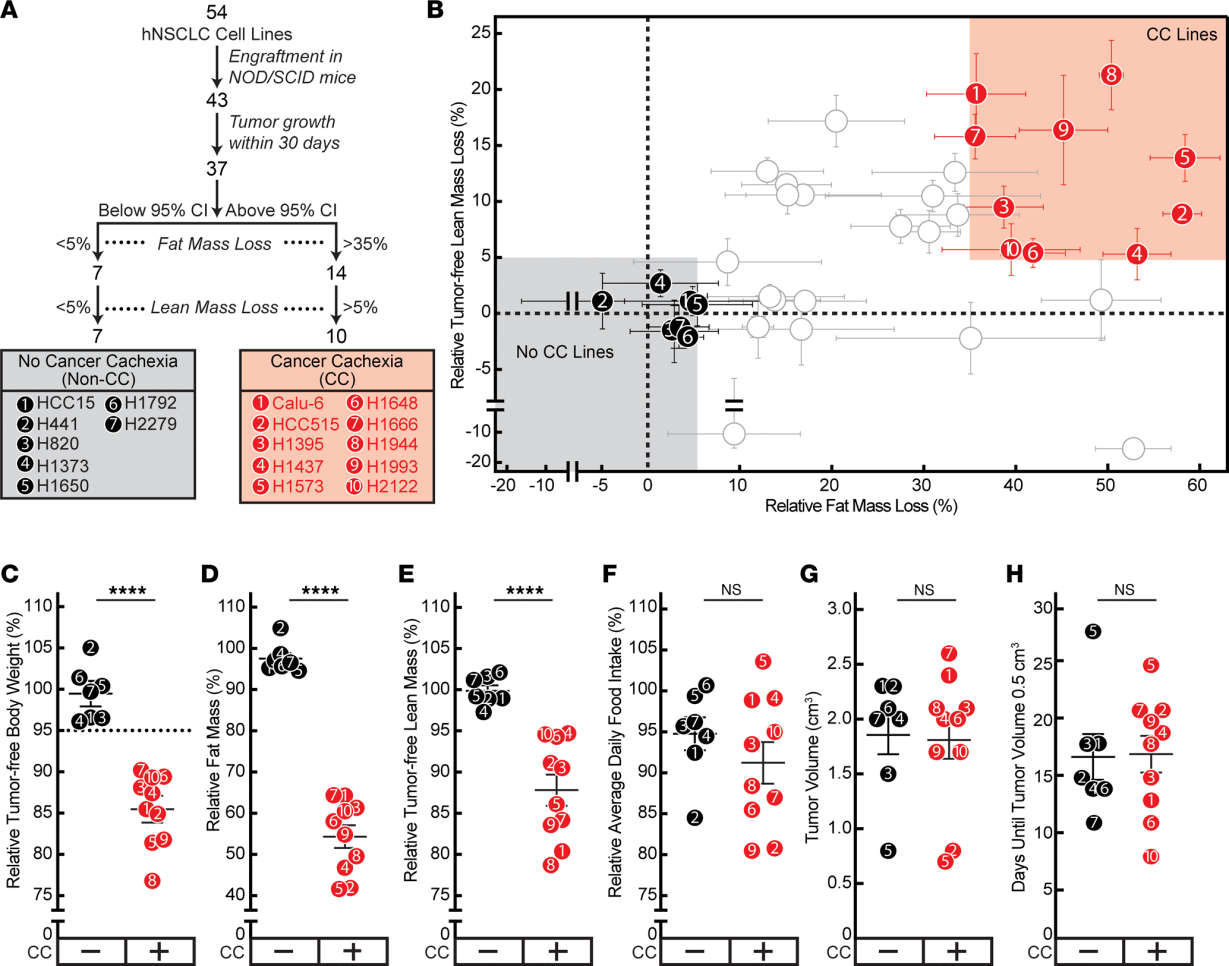
Human NSCLC cachexia screen. (**A**) Schema of CC screen. (**B**–**H**) Identification of human NSCLC lines with and without CC potential. Chow-fed NOD/SCID mice (11- to 12-week-old male mice, *n* = 6 per cohort) were injected s.c. into the right flank with human NSCLC cells for each of the 54 lines. Measurements of relative terminal fat (**B** and **D**) and tumor-free lean (**B** and **E**) mass, tumor-free terminal body weight loss (**C**), average daily food intake (**F**), terminal tumor volume (**G**), and tumor growth time to 0.5 cm^3^ (**H**) were obtained as described in Methods. CC lines (*red circles*) were defined if tumor growth occurred within 30 days, relative fat mass loss was >35%, and relative lean mass loss was >5%. Non-CC lines (*black circles*) were defined if tumor growth occurred within 30 days, relative fat mass loss was <5%, and relative lean mass loss was <5%. Data are shown as mean ± SEM. ****P < 0.0001 was calculated from 2-tailed unpaired *t* test for significant differences in mean value of the indicated parameter between CC and non-CC groups. CC, cancer cachexia.

To identify biomarkers and molecules critical for tumor-associated wasting, we initiated a screen of human NSCLC lines for cachexia-inducing capacity in vivo (Figure 1A). We allotransplanted 54 NSCLC cell lines subcutaneously into NOD/SCID mice and subsequently assessed changes in host body weight, body composition (lean and fat mass), daily food intake, and tumor growth (Supplemental Table 1; supplemental material available online with this article; https://doi.org/10.1172/jci.insight.165419DS1). Out of the 54 NSCLC lines injected into mice, 43 lines grew tumors, with 37 of these lines having tumor engraftment within 30 days of allotransplantation. Of these latter 37 lines, the median fat mass loss was approximately 20% (95% confidence interval [CI], 8%–35%), and median lean mass loss was approximately 5% (95% CI, 1%–10%). We subsequently dichotomized a subset of these 37 lines into 2 primary cohorts: 10 lines that caused CC as defined by induction of relative fat loss >35% (above 95% CI) and lean mass loss >5% (above median) and 7 lines that failed to cause CC as determined by induction of relative fat loss <5% (below 95% CI) and lean mass loss <5% (below median) in the murine hosts (Figure 1B). On average, mice bearing the NSCLC lines that induced CC had ~15% reduction in body weight (Figure 1C), ~40% reduction in fat mass (Figure 1D), and ~13% reduction in lean mass (Figure 1E) compared with the mice bearing NSCLC lines that could not induce CC in vivo. Importantly, there was no statistical difference in food intake (Figure 1F), tumor volumes (Figure 1G), and tumor growth kinetics (Figure 1H) between cohorts. This screen provided clear phenotypic demarcations between lines with and without the capacity to induce CC in vivo, characteristics not explained by differences in food intake or tumor growth.

### Identification of an NSCLC cachexia oncogenotype.

**Figure 2:**
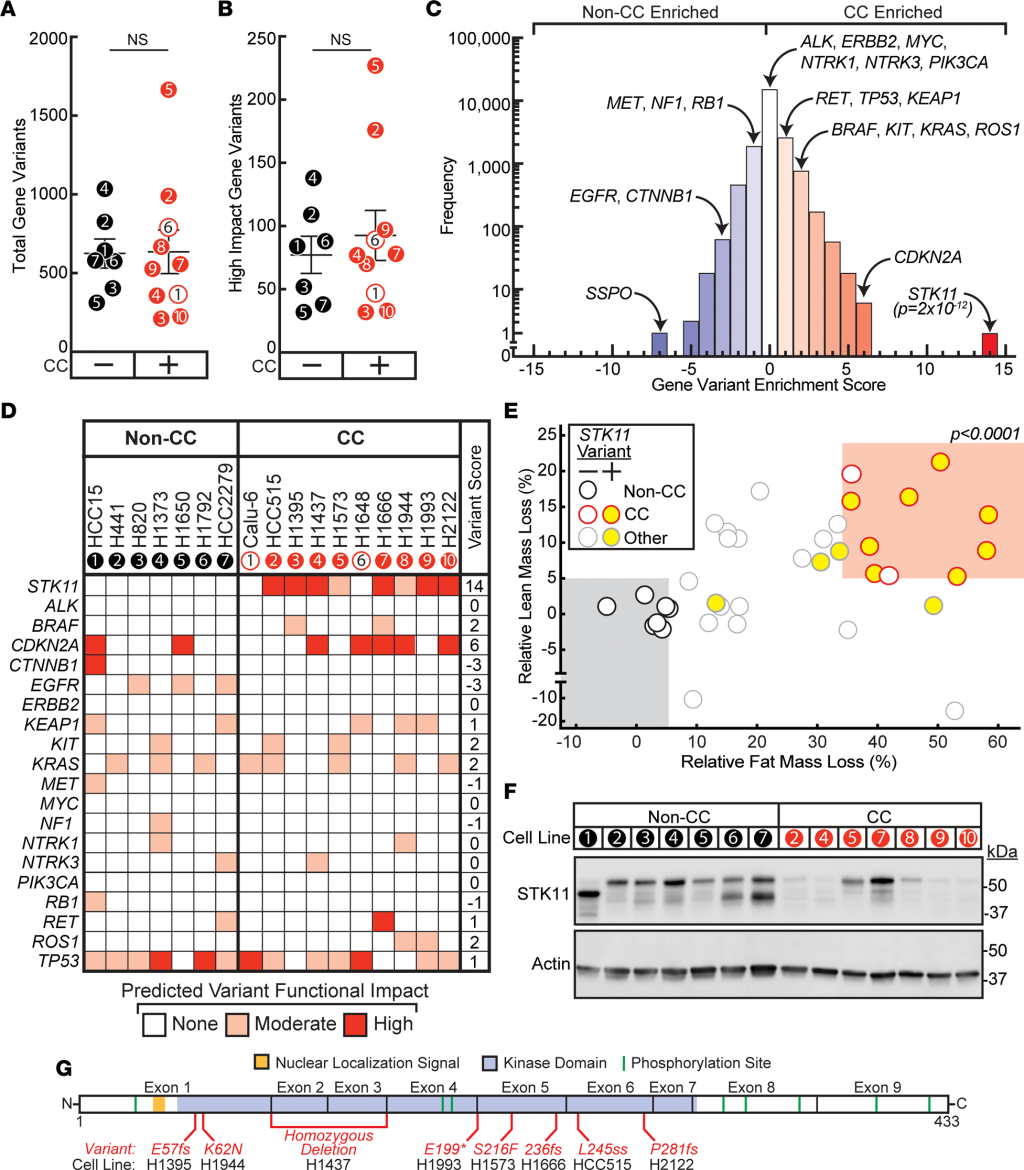
Gene variant analysis of human NSCLC cachexia. (**A** and **B**) Tumor gene variant burden. The tumor mutation burden of human NSCLC lines was evaluated by calculating the frequency of total variant genes in each line (**A**) or total high-impact-variant genes in each line (**B**). (**C**) Gene variant analysis. Histogram of gene variant enrichment score (GVES) of all 21,200 profiled genes as described in Methods. (**D**) Heatmap of gene variants of *STK11/LKB1* and selected co-occurring genes common to NSCLC. (**E**) Identification of non-CC (*black outer circle*), CC (*red outer circle*), and other (*gray outer circle*) lines as defined in the CC screen of 37 NSCLC lines associated with wild-type (*white-filled circle*) or variant (*yellow-filled circle*) *STK11/LKB1*. (**F**) Immunoblot analysis of tumors derived from the indicated cell line with the indicated antibody as described in Methods. (**G**) Schematic of *STK11/LBK1* variants found in screened CC cell lines mapped to kinase domain. *P* was calculated based on unpaired 2-tailed *t* test (**A** and **B**), Gaussian distribution fitted to a histogram constructed from all log-odds ratios with Bonferroni adjustment (**C**), or from multiple logistic regression of *STK11/LKB1* status with relative lean mass loss and relative fat mass loss (**E**).

Having identified distinct CC- and non–CC-inducing cohorts in vivo, we next attempted to associate oncogenotypes with CC potential. Using whole-exome sequencing of the NSCLC lines from our in vivo screen (obtained from JDM and available through COSMIC Cell Line Encyclopedia), we measured the frequency of non-synonymous variants across 21,200 genes in each line. There was no significant difference in average frequency of total gene variants (Figure 2A) or high-impact gene variants (Figure 2B) between CC and non-CC cohorts, suggesting that CC potential is not determined by tumor mutation burden alone. A gene variant enrichment score was next calculated to rank genes by association of variant status with CC potential as described in Methods and presented as a histogram of these scores (Figure 2C). *STK11*, also known as liver kinase B1 (*LKB1*), which encodes a cellular regulator of energy and metabolism through its kinase action on AMPK (5–9), had the greatest positive correlation (*P_adj_* = 2 × 10^–12^) of variant status with CC potential. Figure 2D shows the variants in *STK11/LKB1* and other genes commonly associated with NSCLC development in the context of the CC and non-CC cell lines. In total, 80% of the NSCLC CC lines had an *STK11/LKB1* gene variant compared with 0% of the NSCLC non-CC lines.

We next depicted the tumor lines with *STK11/LKB1* variants in our primary human NSCLC cachexia screen as a function of the relative fat and lean mass loss they promoted in immunodeficient mice (Figure 2E). Of the 37 lines that engrafted tumors within 30 days, 12 lines had an *STK11/LKB1* variant, out of which 11 (92%) induced at least 25% fat mass loss concomitant with lean mass loss when injected into mice. Immunoblot analysis suggested that a majority of tumors derived from representative CC-inducing cell lines demonstrated decreased STK11/LKB1 protein expression when compared with tumors derived from the NSCLC lines that could not induce CC in vivo (Figure 2F). All *STK11/LKB1* variants in the 8 CC-associated human NSCLC lines were mapped to exons 1–7 of the gene containing its kinase domain (Figure 2G). Though the variant in line H1437 was a homozygous deletion, the *STK11/LKB1* variants for all other CC lines were non-synonymous (nonsense and missense) single-nucleotide mutations of 100% allele frequency. The lone exception was line H1666, which had an *STK11/LKB1* variant allele frequency of 57.7%. Overall, this variant analysis of the tumor lines from our NSCLC cachexia screen suggested that non-synonymous variants in *STK11/LKB1* are a potential predictive oncogenotype for CC.

### Evaluation of tumor STK11/LKB1 loss of function in promoting human NSCLC-associated cachexia.

**Figure 3:**
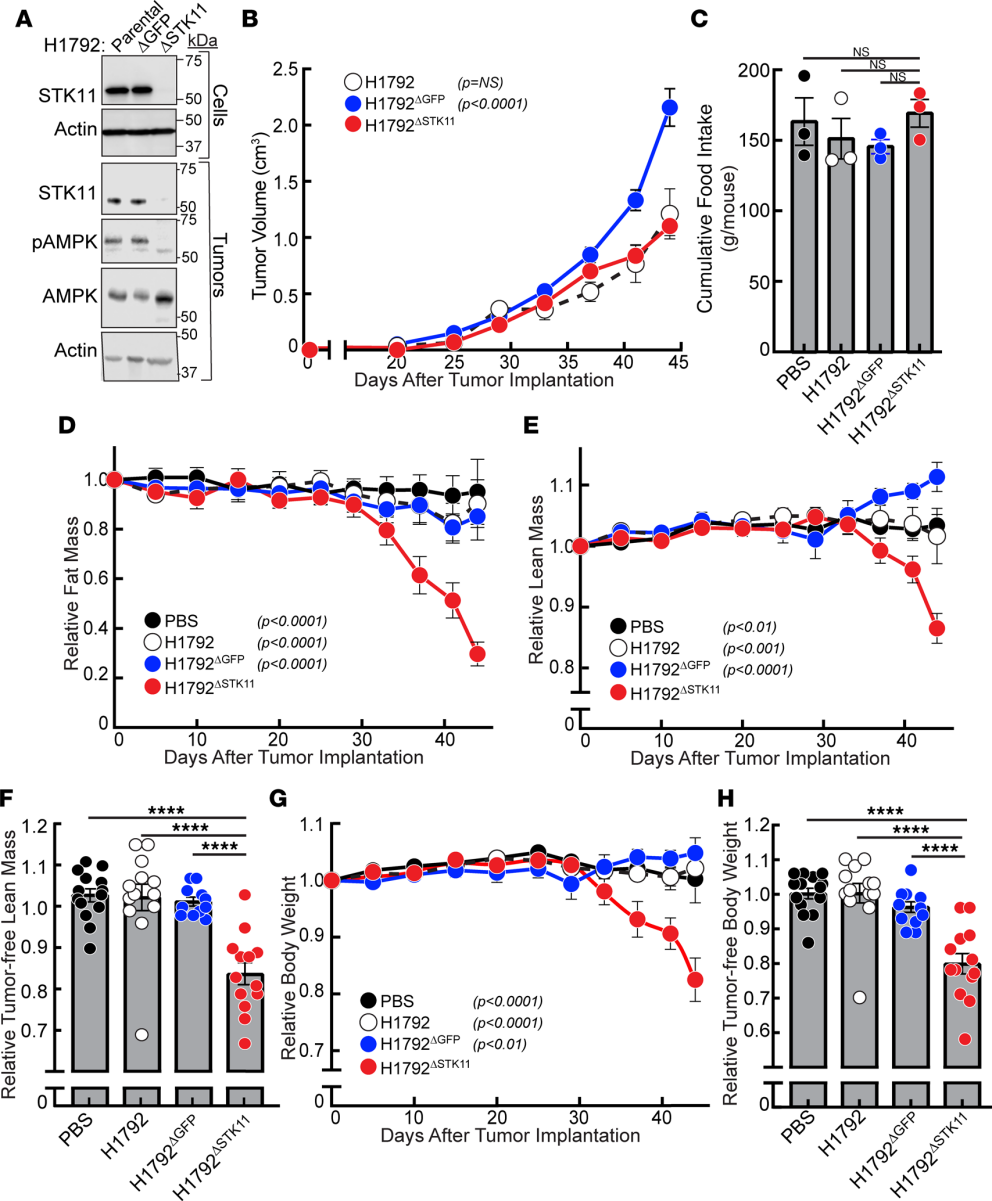
*STK11/LKB1* silencing in a non–cachexia-inducing human NSCLC line. (**A**–**H**) Chow-fed NOD/SCID mice (14–23 week-old male mice, *n* = 11–14 per cohort) were injected s.c. with vehicle (*black closed circles*) or cells from parental H1792 (*black open circles*), H1792^ΔGFP^ (*blue circles*), or H1792^ΔSTK11^ (*red circles*) lines as described in Methods. Longitudinal measurements of tumor volume (**B**), cumulative food intake (**C**), fat mass (**D**), lean mass (**E** and **F**), and body weight (**G** and **H**) were obtained as described in Methods. Injected cells and tumors at sacrifice were processed for immunoblot analysis with the indicated antibodies as described in Methods (**A**). Data are shown as mean ± SEM of the actual measurements (**B** and **C**) or relative to their day 0 values (**D**–**H**) over 3 independent experiments. *P* was calculated using 1-way (**C**, **F**, and **H**) or 2-way (**B**, **D**, **E**, and **G**) ANOVA followed by Dunnett’s multiple-comparison test for significant differences from the H1792^ΔSTK11^ cohort. ****P < 0.001.

After establishing the association between *STK11/LKB1* variants in NSCLC and CC, we next determined if silencing *STK11/LKB1* would convert an NSCLC line incapable of promoting adipose and muscle loss into a CC-inducing line in vivo. Our CC screen identified H1792 as a human lung adenocarcinoma line without CC-inducing potential (<5% fat and 0% lean mass loss) in vivo. The H1792 line harbors wild-type *STK11/LKB1* but co-occurring *TP53* and *KRAS* variants (see Figure 2D). To validate the role of STK11/LKB1 in regulating host wasting, the H1792 NSCLC cell line was infected with lentiviral CRISPR/Cas9 constructs possessing guide RNA directed against the kinase domain of *STK11/LKB1* (H1792^ΔSTK11^) to silence expression of the gene as described in Methods. The parental H1792 cell line was also independently infected with guide RNA targeting *GFP* gene as a control (H1792^ΔGFP^). Immunoblot analysis of the parental and engineered H1792 cell lines demonstrated only decreased STK11/LKB1 protein expression in the H1792^ΔSTK11^ tumor cells compared with H1792^ΔGFP^ and parental H1792 tumor cells (Figure 3A).

**Figure 4:**
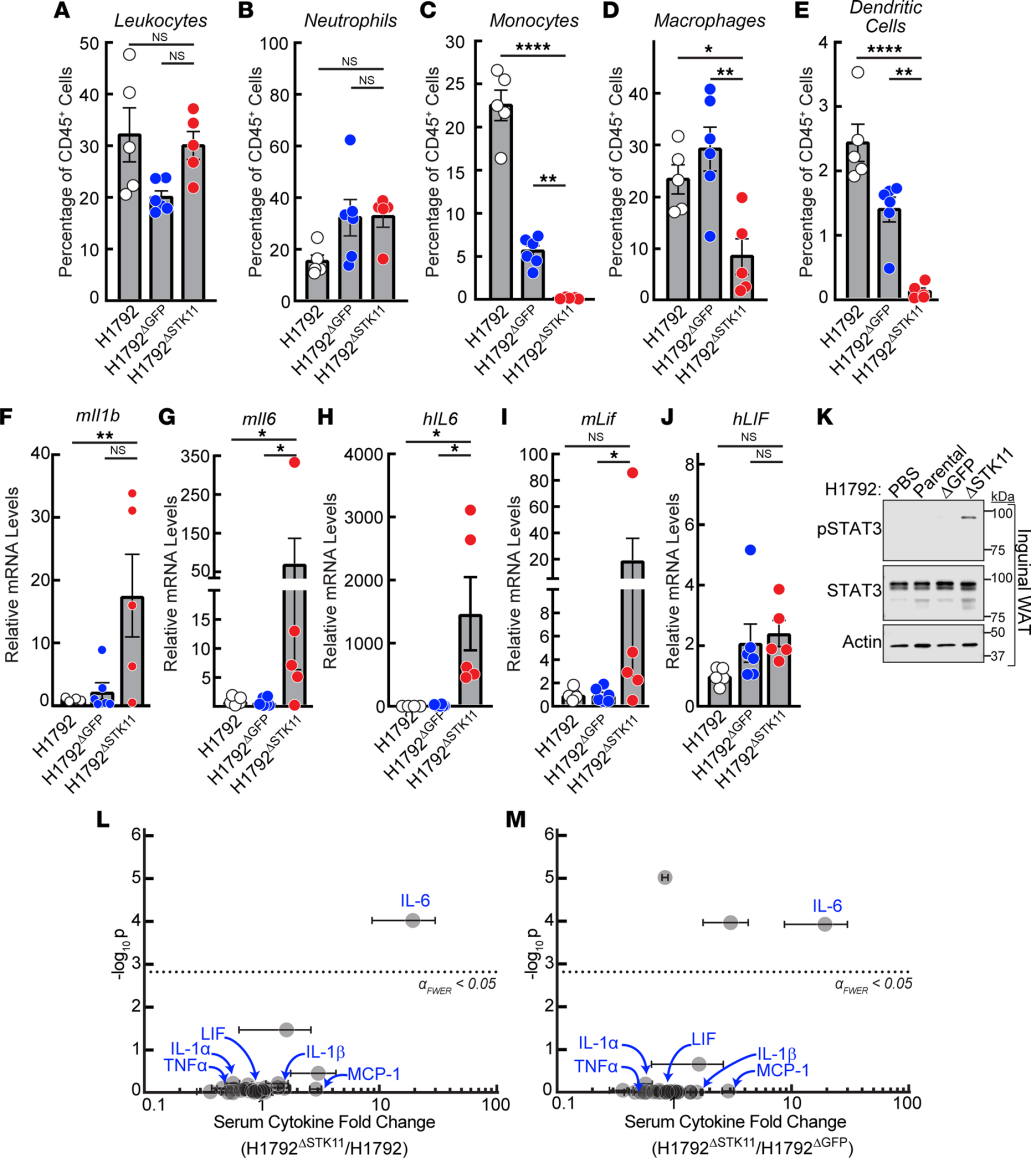
Immunophenotype of *STK11/LKB1* variant human NSCLC tumors inducing cachexia. (**A**–**M**) Tumor, serum, or inguinal white adipose tissue samples at sacrifice from the experiment in Figure 3 were processed for measurement of the levels of tumor-infiltrating myeloid cells (**A**–**E**) by FACS analysis (see Supplemental Figure 1 for gating strategy), tumor mRNA levels relative to H1792 parental cohort of the indicated genes normalized to β*-*actin (**F**–**J**), immunoblot analysis of inguinal white adipose tissue using the indicated antibodies (**K**), or serum cytokine levels (**L** and **M**) as described in Methods. Data are shown as mean ± SEM. *P < 0.05, **P < 0.01, and ****P < 0.0001 based on 1-way (**A**–**J**) or 2-way (**L** and **M**) ANOVA followed by either Dunnett’s (**A**–**J**) or Bonferroni’s (**L** and **M**) multiple-comparison test for significant differences from the H1792^ΔSTK11^ cohort. WAT, white adipose tissue.

Subsequently, we subcutaneously injected cells from the parental H1792, H1792^ΔGFP^, or H1792^ΔSTK11^ lines into NOD/SCID mice and longitudinally assessed tumor growth, body composition, body weight, and food intake. Immunoblot analysis of the malignancies derived from these lines revealed that only the tumors obtained from mice injected with the H1792^ΔSTK11^ cells maintained decreased STK11/LKB1 protein expression with a concomitant decrease in the phosphorylation of its downstream target AMPK compared with the parental H1792 and the H1792^ΔGFP^ tumors (Figure 3A). As shown in Figure 3B, there were no statistically significant differences in tumor volumes derived from the parental H1792 versus H1792^ΔSTK11^ cell lines in vivo, though the H1792^ΔGFP^ control tumors were statistically larger than the other 2 cohorts. The lentivirus-enabled creation of the *STK11/LKB1*-knockout line led to decreased tumor growth compared with the lentivirus-enabled creation of the *GFP* control line in the NOD/SCID immunodeficient model, consistent with the observations of other groups (10). Average cumulative food intake per mouse was not significantly different among the cohorts over the course of 3 independent experiments (Figure 3C). Despite having smaller tumor volumes, mice injected with H1792^ΔSTK11^ cells over time demonstrated a 40%–50% decrease in relative fat mass as compared with control cohorts (Figure 3D). This same experimental mouse cohort injected with *STK11*-silenced tumor cells also displayed a more than 10% decrease in: 1) relative lean mass including tumor (Figure 3E), 2) relative tumor-free lean mass at sacrifice (Figure 3F), 3) relative body weight including tumor (Figure 3G), and 4) relative tumor-free body weight at sacrifice (Figure 3H) compared with control cohorts. Overall, the silencing of *STK11/LKB1* in tumor cells converted a human NSCLC line incapable of inducing cachexia into one that induced cachexia-associated fat and lean mass loss, resulting in approximately 15% body weight loss without affecting food intake and despite having smaller tumor volumes compared with controls.

### Tumor immune microenvironment, systemic, and host tissue changes in mice bearing human STK11/LKB1-silenced tumors.

Previous groups have demonstrated a change in the immune cell composition of microenvironments of *STK11/LKB1*-variant NSCLC tumors (11). To demonstrate whether these changes were present in *STK11/LKB1*-silenced tumors that induced cachexia-associated fat and muscle wasting, we evaluated the percentage of total immune cells, neutrophils, monocytes, macrophages, and dendritic cells in tumors generated from H1792^ΔSTK11^ and control cohorts in the experiments outlined in Figure 3. Flow cytometry analysis of tumor immune cells (using the gating strategy as described in Supplemental Figure 1) demonstrated that although H1792^ΔSTK11^ tumors had similar percentages of leukocytes (CD45^+^) (Figure 4A) compared to tumors derived from the 2 control lines and trended toward having more neutrophils (CD11b^+^Ly6G^+^) when compared with only the control parental tumors (Figure 4B), these tumors displayed significantly fewer monocytes (F4/80^–^Ly6C^hi^) (Figure 4C), macrophages (F4/80^+^Ly6C^–^) (Figure 4D), and dendritic cells (CD11c^+^) (Figure 4E) as a percentage of leukocytes in comparison with the control cohorts, as seen previously (11).

In Figure 4, F–J, and Supplemental Figure 2, quantitative PCR analysis of the above tumors using human and murine primers that distinguished tumor cell (human) and host cell (mouse) intrinsic gene transcripts were used to evaluate expression of CC-associated cyto/chemokines and other molecules associated with *STK11/LKB1* NSCLC gene alterations (4, 11). Expression of the chemokines *Cxcl7* (Supplemental Figure 2C) and *Ccl5* (Supplemental Figure 2D) was significantly elevated, and *Cxcl2* (Supplemental Figure 2A) and *Cxcl12* (Supplemental Figure 2B) trended upward in H1792^ΔSTK11^ tumors compared with control tumors when using mouse primers, concordant with the observed changes in tumor immune cellularity and consistent with host responses. When measuring for changes in levels of cachexia-associated factors, *Il1b* (Figure 4F), *Il6* (Figure 4G), and leukemia inhibitory factor (*Lif*) (Figure 4I) but not *Tnf* (Supplemental Figure 2F) or *Gdf15* (Supplemental Figure 2H) mRNA expression was elevated when using mouse primers, with only *IL6* mRNA expression (Figure 4H) elevated when also using human primers in H1792^ΔSTK11^ tumors compared with tumors from control cohorts. *IL-1β* tumor mRNA levels were undetectable when using human primers across all cohorts. In parallel, multiplex mouse cytokine serum analyses conducted from the above mouse studies identified only IL-6 from the 32 measured cytokines to be significantly increased in the serum from H1792^ΔSTK11^ tumor–bearing mice when compared with serum from mice bearing parental H1792 (Figure 4L) or H1792^ΔGFP^ (Figure 4M) control tumors.

Previously, we demonstrated that cachexia-associated adipose loss induced by IL-6 family cytokines is associated with white adipose tissue STAT3 phosphorylation (4, 12). Consistent with the cachexia phenotype, mice bearing H1792^ΔSTK11^ tumors demonstrated increased STAT3 activation in adipose tissue compared with control cohorts (Figure 4K). We also previously showed that cachexia-associated adipose loss induced by IL-6 family cytokines is suppressed with systemic JAK inhibition that leads to a decrease in host tissue STAT3 activation (4, 12). In light of our previous data associating increased IL-6 family pro-inflammatory cytokine levels with CC induced by tumor *STK11*/*LKB1* loss of function, we next determined if the JAK inhibitor ruxolitinib could block cachexia-induced adipose wasting in vivo observed with allotransplantation of the parental human NSCLC *STK11*-variant H1573 line into mice. Administration of ruxolitinib had no effect on tumor growth kinetics (Supplemental Figure 3A), but it significantly suppressed fat loss (Supplemental Figure 3B) and adipose STAT3 phosphorylation (Supplemental Figure 3C) induced in the context of *STK11*-variant H1573 tumors. Collectively, these studies verified that tumors which induce cachexia in vivo because of *STK11/LKB1* silencing display changes in their tumor immune landscapes similar to observations made by other groups in NSCLC tumors enriched for *STK11/LKB1* alterations (11). Furthermore, our studies demonstrated that *STK11/LKB1* alterations promote local and systemic increases in pro-inflammatory cytokines associated with signaling changes in host tissues that resemble the ones previously observed with cachexia-associated wasting (4, 12–18).

### Role of tumor STK11/LKB1 loss of function in promoting CC development in an immunocompetent CRC murine model.

**Figure 5:**
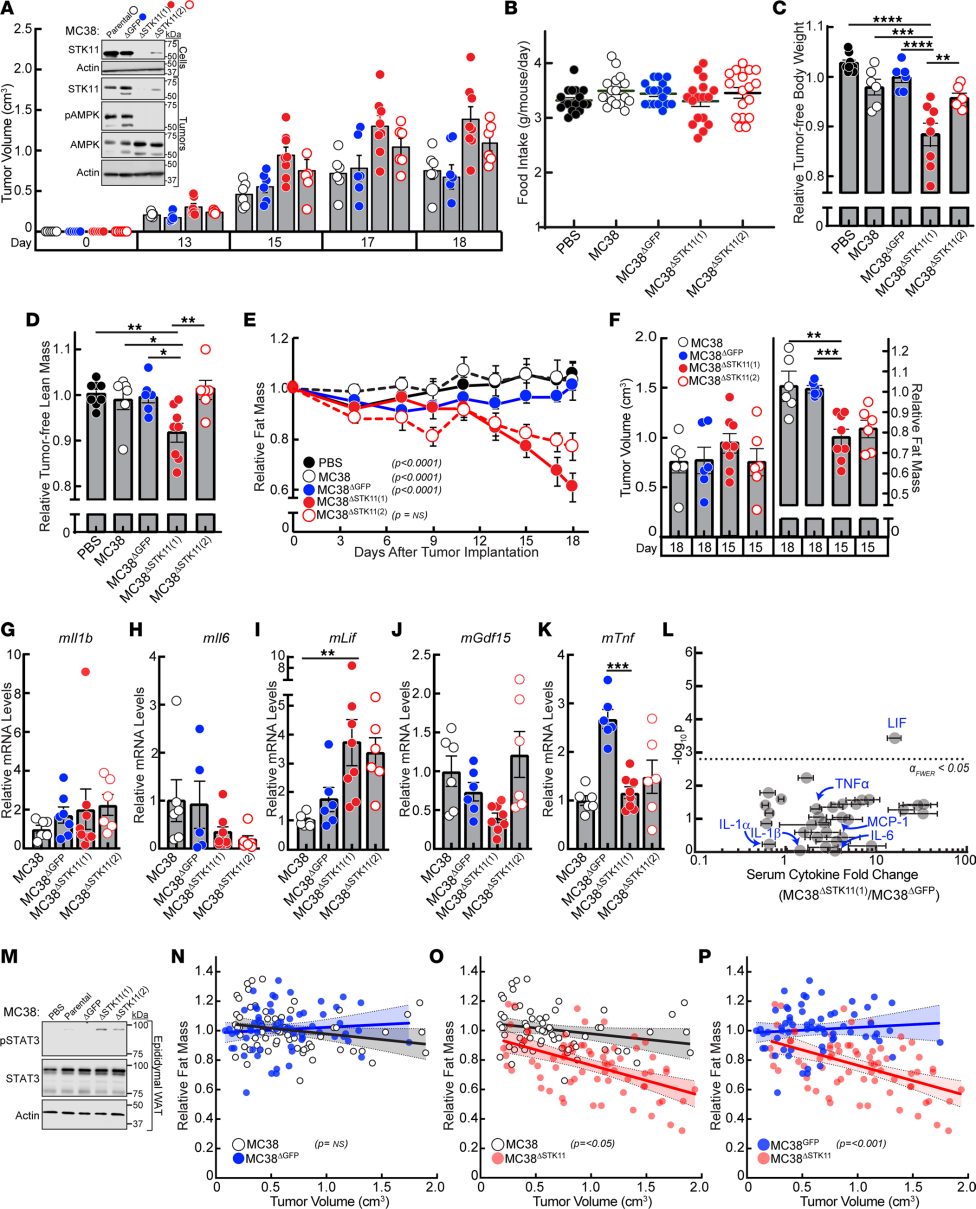
*STK11/LKB1* silencing in a non–cachexia-inducing murine CRC line. (**A**–**M**) Chow-fed C57BL/6J mice (12-week-old male mice, *n* = 6–8 per cohort) were injected s.c. with vehicle (*black closed circles*) or cells from the parental MC38 (*black open circles*), MC38^ΔGFP^ (*blue circles*), or 2 different MC38^ΔSTK11^ (*red closed and open circles*) lines as described in Methods. Longitudinal measurements of tumor volume (**A** and **F**), daily food intake (**B**), tumor-free body weight at sacrifice (**C**), tumor-free lean mass at sacrifice (**D**), and fat mass (**E** and **F**) were obtained as described in Methods. Tumor or serum samples at sacrifice were processed for measurement of the levels of tumor mRNA levels relative to MC38 parental cohort of the indicated genes normalized to *β-actin* (**G**–**K**) or serum cytokine levels (**L**) as described in Methods. Injected cells (**A**, inset), tumors at sacrifice (**A**, inset), and epididymal/gonadal white adipose tissue at sacrifice (**M**) were processed for immunoblot analysis with the indicated antibodies as described in Methods. Data are shown as mean ± SEM of the actual measurement (**A** and **B**) or relative to their day 0 values (**C**–**F**). *P < 0.05, **P < 0.01, ***P < 0.001, and ****P < 0.0001 based on 2-way (**A** and **E**) or 1-way (**B**–**D** and **F**–**L**) ANOVA followed by a Tukey’s (**A** and **E**), Dunnett’s (**B**–**D** and **F**–**K**), or Bonferroni’s (**L**) multiple-comparison test for significant differences from the MC38^ΔSTK11(1)^ cohort. (**N**–**P**) Chow-fed C57BL/6J mice, 12- (*n* = 8), 14- (*n* = 8), and 18- (*n* = 6) week-old males, were injected s.c. with cells from the parental MC38 (*black open circles*), MC38^ΔGFP^ (*blue circles*), or MC38^ΔSTK11^ (*red closed and open circles*) lines as described in Methods. Longitudinal measurements of tumor volume and fat mass were plotted for each cohort. Linear regression analysis was conducted to determine the association of tumor size and fat mass. Data are shown as dot plots with regression line and 95% confidence band.

The above studies validated the role of STK11/LKB1 in NSCLC CC development in immunodeficient mice. We next attempted to generalize the importance of STK11/LKB1 to CC development with the use of a different primary cancer using syngeneic, immunocompetent in vivo models. The well-established non-cachexia murine MC38 CRC cell line has been shown to have variants in multiple genes but not in *STK11*/*LKB1* or *KRAS* (19, 20). We separately silenced *STK11*/*LKB1* in 2 cohorts of parental MC38 cells using lentiviral CRISPR/Cas9 techniques — MC38^ΔSTK11(1)^ and MC38^ΔSTK11(2)^ — to create 2 new isogenic lines. A control cell line was infected with guide RNA targeting GFP (MC38^ΔGFP^). Immunoblot analysis verified that MC38^ΔSTK11(1)^ cells had complete suppression of STK11/LKB1 protein expression (Figure 5A, inset). We also proceeded to study the MC38^ΔSTK11(2)^ cell line, which had substantial but not complete suppression of STK11/LKB1 protein expression.

We injected all of these cell lines into C57BL/6J mice and followed these models for changes in tumor growth, body weight, body composition, and food intake. All MC38 lines formed measurable tumors at day 13 after tumor transplantation (Figure 5A). Despite all average tumor volumes being statistically equivalent across cohorts and time points, there was a trend toward increasing tumor volumes when *STK11*/*LKB1* was completely silenced. These differences in growth kinetics of NSCLCs with altered tumor *STK11/LKB1* expression in immunocompetent hosts was similarly demonstrated in a recent study (10). Immunoblot analysis of all tumors demonstrated decreased STK11/LKB1 protein expression in MC38^ΔSTK11(1)^ and MC38^ΔSTK11(2)^ tumors compared with control tumors (Figure 5A, inset). As expected, decreased STK11/LKB1 expression was also associated with a decrease in AMPK phosphorylation. There was no obvious difference in daily food intake between all cohorts (Figure 5B). MC38^ΔSTK11(1)^ tumors caused nearly 10% weight loss in mice, with no weight loss induced in mice injected with parental MC38 or MC38^ΔGFP^ cells (Figure 5C). This weight loss in the MC38^ΔSTK11(1)^ mice was associated with an approximately 7% loss in muscle/lean mass compared with control cohorts (Figure 5D) and an approximately 40% loss in fat mass (Figure 5E). Microscopic analysis of adipocyte cross-sectional areas taken from epididymal white adipose tissue (Supplemental Figure 4A) and cross-sectional myofiber areas taken from the gastrocnemius muscle (Supplemental Figure 4D) correlated with the ECHO MRI findings and weight loss. Specifically, there was an approximately 75% reduction in adipocyte cross-sectional area (Supplemental Figures 4, B and C) and an approximately 25% reduction in myofiber cross-sectional area (Supplemental Figures 4, E and F) in tissues from mice bearing cachexia-inducing *STK11*-silenced MC38 tumors when compared with mice bearing non–cachexia-inducing MC38 parental tumors. This extent of muscle fiber change was similarly observed by other groups that also used a syngeneic CC cachexia model (21–23). Despite incomplete suppression of STK11/LKB1 protein expression in the MC38^ΔSTK11(2)^ line, tumors derived from these cells still induced an approximately 20% reduction in fat mass in vivo (Figure 5E). However, there were no significant changes in body weight (Figure 5C) or lean mass (Figure 5D) within this cohort.

Assessment of mouse mRNA changes in the tumors of this experiment demonstrated that *Lif* mRNA expression (Figure 5I) was significantly increased whereas *Il6* mRNA expression (Figure 5H) and those of *Il1b* (Figure 5G), *Tnf* (Figure 5K), and *Gdf15* (Figure 5J) were statistically unchanged in MC38^ΔSTK11^ tumors compared with control tumors. Multiplex cytokine serum analyses conducted from this study also only identified LIF from the 32 measured cytokines to be significantly increased in the serum from MC38^ΔSTK11(1)^ tumor-bearing mice when compared with serum from mice bearing MC38^ΔGFP^ control tumors (Figure 5L). Taken together, silencing of *STK11/LKB1* in this colon cancer line was associated with local and systemic elevation of LIF, another IL-6 family pro-inflammatory cytokine associated with CC development in colon cancer murine models (13, 16, 24). As anticipated, only adipose tissue from mice bearing *STK11*/*LKB1*-mutant MC38 tumors demonstrated increased STAT3 phosphorylation, a host tissue response observed in multiple CC murine models driven by IL-6 family cytokine signaling (Figure 5M).

To ensure that the increased wasting observed in the setting of *STK11*/*LKB1* loss-of-function variant tumors in immunocompetent hosts was not secondary to differential tumor size, we compared fat loss at the day 15 time point when the *STK11*/*LKB1*-silenced tumors had similar average tumor volumes compared to the day 18 time point for the control cohorts (Figure 5F). Although there were no statistical differences in tumor volume when comparing those time points, mice transplanted with the MC38^ΔSTK11^ lines still displayed significant fat loss compared with the control cohorts.

To further ensure that the increased wasting observed in the setting of *STK11*/*LKB1* loss-of-function variant tumors in immunocompetent hosts was not secondary to differential tumor size, we also analyzed fat loss as a function of tumor volume using linear regression analysis from 3 combined experiments. There was no difference in fat loss when comparing mice transplanted with MC38 parental lines to mice transplanted with MC38^ΔGFP^ lines across all tumor volumes (Figure 5N). Only mice transplanted with the MC38^ΔSTK11^ cell line demonstrated a significant decrease in fat mass across similar tumor volumes when compared with mice transplanted with MC38 parental (Figure 5O) or MC38^ΔGFP^ (Figure 5P) lines. Together these findings demonstrate that *STK11/LKB1* genetic loss is sufficient to induce CC across multiple cancer types and different host backgrounds.

### Validation of STK11/LKB1 variants as a biomarker for NSCLC-associated patient weight loss at cancer diagnosis.

**Figure 6:**
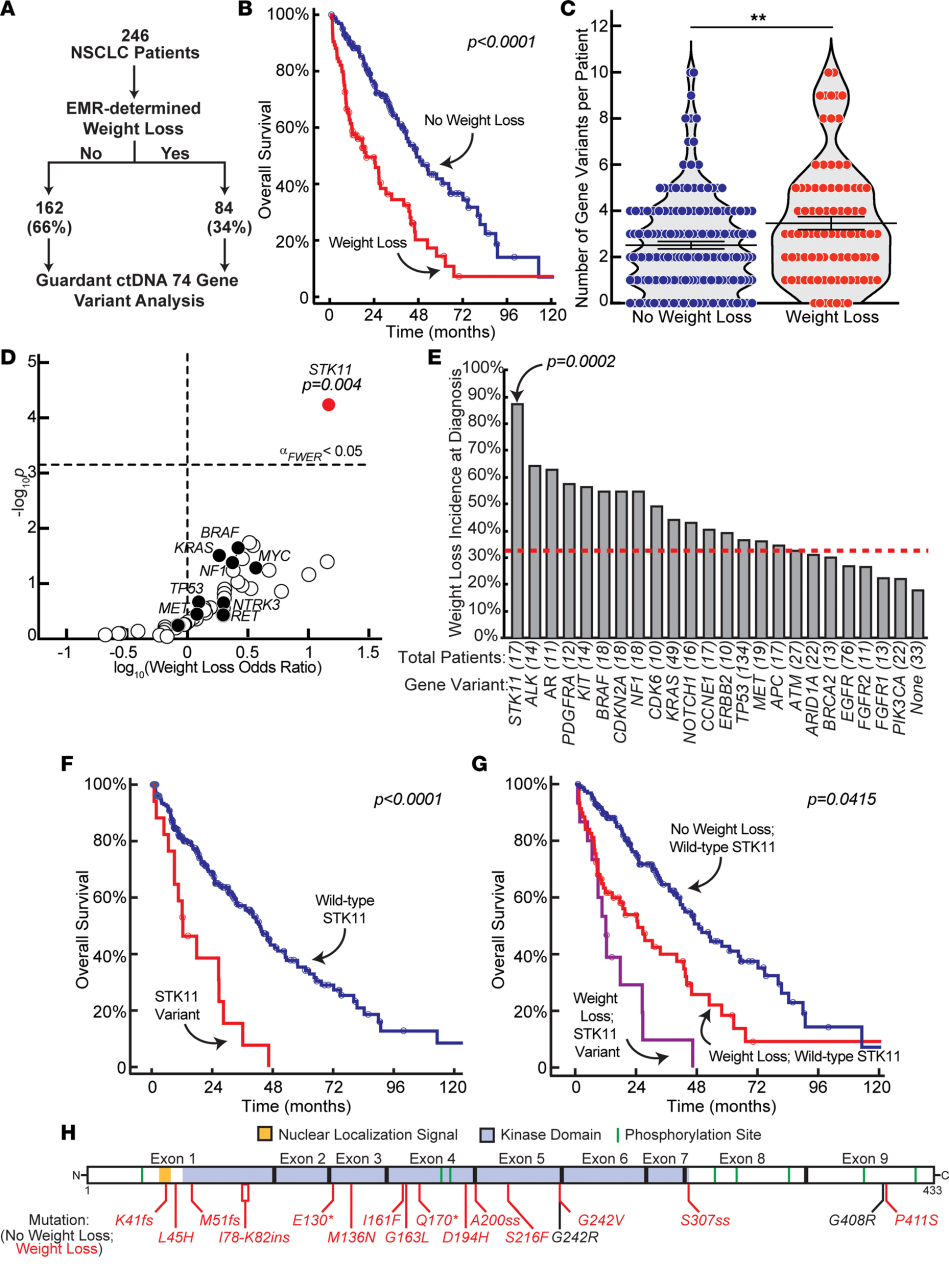
Gene variant analysis of ctDNA from human patients with NSCLC. (**A**) Schema for validation of Guardant gene variant association with NSCLC-associated weight loss at patient cancer diagnosis. (**B**) Overall survival comparison of the 246 patients with NSCLC dichotomized by no weight loss or weight loss at diagnosis. (**C**) Number of tumor cell gene variants (74 genes evaluated) for each of the 246 NSCLC patients. (**D**) Odds ratio for enrichment of gene variants in cancer-associated weight loss patients with NSCLC. (**E**) Incidence of cancer-associated weight loss at diagnosis for genes with variants in ≥10 patients. (**F** and **G**) Overall survival curve of NSCLC patients grouped by *STK11/LKB1* variant status (**F**) or both *STK11/LKB1* and cancer-associated weight loss status (**G**). (**H**) Schematic of *STK11/LBK1* variants found in NSCLC patient ctDNA colored by non–cancer-associated (*black*) or cancer-associated weight loss (*red*). **P < 0.01 or *P* calculated by log-rank tests (**B**, **F**, and **G**), unpaired 2-tailed *t* test (**C**), odds ratio *z* score with Bonferroni adjustment (**D**), or χ^2^ test with Bonferroni adjustment (**E**).

To create a data set that could be used to validate our human NSCLC cachexia screen, we procured a list of 246 patients with NSCLC who had blood collected for evaluation of variants in 74 genes (including *STK11/LKB1*) with the Guardant360 Companion Diagnostic (CDx) circulating tumor DNA (ctDNA) assay as part of an IRB-approved protocol (Figure 6A). All ctDNA analyses for the cohort were conducted within the past 2–3 years, supporting homogeneity in treatment protocols among patients and thus limiting the potential influence of treatment heterogeneity on ctDNA variants identified. Some patients had multiple longitudinal blood collections submitted for ctDNA analysis. As a consequence, we only included the test result at the time of or closest to the patient’s original NSCLC diagnosis. These restrictions led to a final database of 246 patients with ctDNA mutational analysis. For studies on overall survival among other endpoints, we also had long enough follow-up for evaluation of all 246 patients in the cohort. In total, nearly 85% of patients in this cohort had locally advanced or advanced NSCLC (Supplemental Table 2). There were equal numbers of men and women in the overall cohort. Median patient age at diagnosis was approximately 66. The cohort had an expected distribution of squamous and non-squamous tumor histologies. Approximately 73% of the cohort were non-Hispanic White, and the rest were Black, Hispanic, or Asian. Data on overall survival and other clinical parameters for patients were obtained from the University of Texas Southwestern Harold Simmons Comprehensive Cancer Center Tumor Registry.

This data set was subsequently used to identify associations between tumor gene variants and the presence of cancer-induced weight loss at original cancer diagnosi*s*. The method of identifying patients with cancer-associated weight loss at diagnosis is described forthwith. Briefly, without knowledge of the Guardant data, 1 primary and 1 secondary reviewer separately identified the presence or absence of nonpurposeful weight loss at cancer diagnosis. Patients are routinely weighed as a part of each office visit in our health system, with measurements documented in the electronic medical record. Using these data, we incorporated the validated international consensus definition of CC and pre-cachexia when describing our cohort of patients with cancer-associated weight loss (25, 26). Specifically, we considered cancer-associated weight loss to be present if patients met the criteria of having unintentional weight loss > 5% within 6 months preceding cancer diagnosis if they had a body mass index ≥ 20 kg/m^2^ or unintentional weight loss > 2% with body mass index < 20 kg/m^2^ (both cachexia) or weight loss up to 5% or 2% for the respective body mass indices (pre-cachexia). When multiple measures of weight were available in the pretreatment period, a consistent weight decrease was required for a patient to be classified as having weight loss. Patients with stable weight, weight gain, or purposeful weight loss were classified as not having weight loss. This weight loss was determined numerically if we could identify weights taken prior to an NSCLC diagnosis. Alternatively, a patient was deemed as having wasting if significant (pre-cachexia or cachexia consensus definition level) weight loss was patient reported in a review of symptoms by at least 2 of the following medical professionals — physician (medical oncologist, pulmonologist, radiation oncologist, surgical oncologist, emergency room, etc.) — at the time of diagnosis and/or by at least 1 other medical provider — nurse, nutritionist, dietitian, advanced practice providers, among others. If the 2 reviewers had a discrepancy in their determination of patient weight loss, a third reviewer would adjudicate the outcome.

As anticipated from our prior studies (2), one-third of patients in the cohort had cancer-associated weight loss at diagnosis (Figure 6A). Patient characteristics of the entire cohort as a function of weight loss status are presented in Supplemental Table 2. Having cancer-associated weight loss at diagnosis was associated with a decrease in overall median survival (~20 months) compared with those patients without this weight loss at diagnosis (47 months) (*P* < 0.001) (Figure 6B), findings compatible with our larger cohort study with a similar patient population (2). Survival was calculated from time of cancer diagnosis to death, with patients censored at the time of last clinic visit (if lost to follow-up) or end of study period.

After we determined whether each patient exhibited cancer-associated weight loss, we determined whether each patient had a non-synonymous variant in any of 74 evaluated genes. Figure 6C shows that NSCLC patients with weight loss had a small, but statistically significant, increase in tumor total mutation burden compared with the NSCLC patients without weight loss. Among the 74 genes that were evaluated, variants in *STK11/LKB1* were the only ones that were significantly associated with cancer-induced weight loss at diagnosis (Figure 6D). The odds ratio for this association was approximately 17 (*P_adj_* = 0.004). Thirty-four percent of all the patients with NSCLC in this data set had cancer-associated weight loss, but nearly 90% of patients with an *STK11/LKB1* variant tumor had cancer-associated weight loss (*P* = 0.0002) (Figure 6E). Contrarily, no variants of the other 73 genes correlated with cancer-associated weight loss at cancer diagnosis.

We next mapped all the *STK11/LKB1* variants associated with cancer-induced weight loss. As shown in Figure 6H, most variants were within the *STK11/LKB1* kinase domain. These included nonsense and missense mutations. As expected, *TP53* and *KRAS* mutations were most frequently co-occurring with the *STK11/LKB1* variants (Supplemental Figure 5). However, unlike *STK11/LKB1*, neither gene nor their variants were statistically associated with weight loss at diagnosis (see Figure 6, D and E). Critically, patients with *STK11/LKB1*-variant tumors had reduced overall survival compared with patients with no *STK11/LKB1*-variant tumors (Figure 6F). Furthermore, within the patient cohort with weight loss at diagnosis, those with *STK11/LKB1*-variant tumors had significantly reduced overall survival compared with patients with weight loss but no *STK11/LKB1*-variant tumors (Figure 6G). There were too few patients with an *STK11/LKB1*-variant tumor without weight loss (*n* = 2) to do regression analysis with this cohort. Ultimately, the presence of *STK11/LKB1* ctDNA gene variants was highly associated with cancer-associated weight loss at NSCLC diagnosis and portended the worst overall survival among all combination of cohorts.

## Discussion

Despite being recognized over 2,000 years ago by Hippocrates for its severe wasting phenotype (27), the molecular mechanisms driving cachexia development remain elusive. Through an unbiased CC screen of 54 human NSCLC lines, we demonstrated a significant positive association between CC and variants in the gene *STK11/LKB1* (Figure 2E). Loss of human or murine tumor *STK11/LKB1* function led to cachexia-associated adipose and lean mass loss, resulting in significant weight loss (Figures 3H and 5C) in immunodeficient and immunocompetent mice, respectively. CC wasting in mice with *STK11*/*LKB1*-variant tumors was associated with significant alterations in tumor microenvironment immune cell repertoire, concomitant amplification of local and systemic pro-inflammatory cytokines, and host adipose STAT3 activation. Analysis of ctDNA from 246 NSCLC patients validated the association between cancer-associated weight loss at tumor diagnosis and *STK11/LKB1* variants (Figure 6, D and E). These findings are clinically relevant in light of the number of cancers driven by mutations in *STK11*/*LKB1* that are found spontaneously or in the hereditary *STK11*/*LKB1* deficiency of Peutz-Jeghers syndrome (28). Although there are no case series, multiple case reports have documented weight loss in patients who have developed a cancer in the setting of Peutz-Jeghers syndrome (29, 30). Overall, our work proposes variants in tumor *STK11*/*LKB1* as predictive/prognostic biomarkers for CC that also have a parallel biologic role in the syndrome’s development.

It is not surprising that a screen attempting to associate gene variants with a known immunometabolic syndrome, in fact, identified mutations in a gene responsible for concomitantly modulating cell-intrinsic energy metabolism and tumor immune microenvironments. *STK11*/*LKB1* is a kinase that acts upon the master metabolic regulator AMPK and other AMP-related kinases while permitting immune cell changes in tumor microenvironments when mutated in tumor cells (11, 31). Loss of tumor *STK11/LKB1* function not only decreased the phosphorylation/activation of its downstream target AMPK but also was associated with a change in tumor immune composition characterized by decreased monocytes, macrophages, and dendritic cells within the tumor microenvironment as also observed by others (11). These immune cellular changes in *STK11/LKB1* loss-of-function tumors were accompanied by increased mRNA expression and serum concentrations of multiple pro-inflammatory cytokines (IL-1β, IL-6, and LIF). As previously observed by our group and others, systemic elevation of these cytokines is associated with adipose/muscle wasting and anorexia in multiple CC models (4, 12–17). Concordant with these overall findings and the role of these cytokines in CC-associated host tissue wasting, the adipose from the CC models in these current studies demonstrated increased STAT3 phosphorylation/activation when compared with adipose taken from mice without CC. The phosphorylation of STAT3 in adipose from IL-6 family cytokine signaling supports adipocyte browning and lipolysis, resulting in adipose wasting (4, 12, 15). These observations may explain why JAK inhibitors suppressed cachexia-associated adipose wasting in *STK11/LKB1*-deficient tumors. We plan to use single-cell techniques to identify the interactions between the tumor cells and the immune cells of the microenvironments to demonstrate how a tumor-intrinsic loss of function mutation in *STK11/LKB1* drives an immune response conducive to host wasting.

Having identified *STK11*/*LKB1* as a gene variant that supports CC development also provides new opportunities to identify *STK11*/*LKB1* immune and metabolic influences on tumor and host biology. As a first approach, it will be important to demonstrate that STK11/LKB1 is acting through AMPK or its other substrates to induce the changes in the tumor microenvironment potentially supportive of adipose and muscle wasting. Leveraging the isogenic lines with and without the ability to induce cachexia generated in this study provides a controlled system to interrogate how altered energy sensing in tumors elicits a host immune response driving CC independent of contributions from tumor growth or food intake. This altered innate immune response in patients with NSCLC *STK11/LKB1* variants is associated with resistance to standard immuno-oncology (IO) agents that rely primarily on an adaptive immune response (11, 32). Patients with NSCLCs with loss-of-function *STK11/LKB1* variants are thought to have reduced overall survival secondary to IO resistance when compared with patients whose tumors do not possess these mutations. It is possible, however, that the reduced survival may also be secondary to the significant cancer-related weight loss associated with *STK11/LKB1* mutation status. All the above findings suggest that the same mechanisms contributing to *STK11*/*LKB1*-mutant tumor resistance to IO, including pro-inflammatory and immune cascades, may be contributing to cachexia development, or vice versa. The suppression of cachexia with JAK inhibition may thus correlate with improved outcomes. We currently are conducting a cachexia trial evaluating JAK inhibition concurrent with standard-of-care IO-chemotherapy regimens in patients with advanced NSCLC (ClinicalTrials.gov NCT04906746). Finally, we are in parallel evaluating the secretomes of the CC-inducing tumors to determine if new soluble ligands are being created with loss of *STK11*/*LKB1* function that act directly on adipose and/or muscle to promote cachexia.

With their generalized importance in supporting cell transformation, *STK11*/*LKB1* mutations can represent a surrogate in identifying patients who will develop cachexia across multiple primary cancers. These findings may not be limited to NSCLC and CRC but also other cancers including hepatocellular carcinoma that are also associated with alterations in *STK11*/*LKB1*. At this time, it appears mutations in *STK11*/*LKB1* are present in 10%–15 % of NSCLC and 3% of all solid tumors (33). We expect that even for early-stage disease, the presence of an *STK11*/*LKB1* tumor variant should galvanize the patient’s multidisciplinary team to emphasize supportive care measures focused on nutrition, quality of life, and other metrics before significant wasting manifests. Use of the presence of a tumor *STK11*/*LKB1* mutation will also permit cachexia trials to enrich for patients who may most benefit from a therapeutic intervention. Our clinical data demonstrated that one-third of the 246 NSCLC patients assessed in a validation cohort had cachexia-associated weight loss at diagnosis. Of these 84 patients with cachexia, 20% of their tumors had a variant in *STK11*/*LKB1*. These data not only support the significant association of *STK11*/*LKB1* with CC but also suggest that there could be other gene changes contributing to NSCLC CC-associated weight loss. Furthermore, with the high probability that different primary cancers may have other genetic changes dictating cachexia development, we are screening multiple cancers associated with CC and their respective mutation profiles to identify potentially novel tumor-intrinsic genetic drivers of the wasting syndrome.

In summary, our work supports the use of tumor *STK11/LKB1* loss of function as a CC biomarker with predictive and prognostic value. This knowledge will permit enrollment of patients with early cachexia into clinical trials optimizing the potential of CC therapies to suppress additional wasting. Our studies also provide CC treatment paradigms by targeting the tumor microenvironment or molecules downstream of STK11/LKB1, with the goal of improving patient survival and quality of life.

## Methods

Additional information can be found in the Supplemental Methods.

### Materials.

Detailed material information is listed in Supplemental Table 3.

### Buffer and culture medium.

Buffer A contained 10 mM Tris-HCl (pH 6.8), 100 mM NaCl, 1% (*w/v*) SDS, 1 mM EDTA, 1 mM EGTA, Phosphatase Inhibitor Cocktail Set I and Set II, and Protease Inhibitor Cocktail. For immunoblotting, blocking buffer contained 5% (*w/v*) nonfat powdered milk in PBS-Tween (PBS-T), primary antibodies were diluted in a buffer containing 5% (*w/v*) BSA in PBS-T, and secondary antibodies were diluted in the blocking buffer. The 5× loading buffer for SDS-PAGE contained 250 mM Tris-HCl (pH 6.8), 10% sodium dodecyl sulfate, 25% glycerol, 5% β-mercaptoethanol, and 0.2% bromophenol blue. Medium A was RPMI 1640 supplemented with 5% (v/v) fetal bovine serum (FBS), 100 units/mL penicillin, and 100 μg/mL of streptomycin. Medium B was DMEM, high glucose, supplemented with 100 units/mL penicillin and 100 μg/mL of streptomycin. Medium C was medium B supplemented with 5% (v/v) FBS. Medium D was medium B supplemented with 10% (v/v) FBS.

### Cell culture.

Stock cultures of all human NSCLC cell lines (gift from University of Texas Southwestern Medical Center), HEK293T cells (ATCC), and MC38 murine tumor cells (Kerafast) were maintained in monolayer culture at 37°C in 5% CO_2_ in medium A, D, or C, respectively. Each cell line was propagated, aliquoted, and stored under liquid nitrogen. Aliquots of these cell lines were passaged for less than 4 weeks to minimize genomic instability prior to injection into mice. Every 6 months, the cell lines were tested for *Mycoplasma* contamination using the MycoAlert *Mycoplasma* Detection Kit.

### Generation of knockout mammalian cell lines using CRISPR/Cas9.

Monoclonal H1792 and MC38 cell lines harboring genetically silenced *STK11* were derived by lentiviral delivery of lentiCRISPRv2-puro CRISPR/Cas9 knockout constructs. A single guide RNA (sgRNA) directed to human or mouse *STK11* (Δ*STK11*) or to *GFP* as nontargeting control (Δ*GFP*) were subcloned into the *Bsm*BI site of lentiCRISPRv2-puro vector (Addgene plasmid 52961) using the following pairs of annealed oligonucleotides for GFP (forward 5′-CACCGGTGAACCGCATCGAGCTGA-3′; reverse 5′-AAACTCAGCTCGATGCGGTTCACC-3′), human STK11 (forward 5′-CACCGTTGCGAAGGATCCCCAACG-3′; reverse 5′-AAACCGTTGGGGATCCTTCGCAAC-3′), and mouse STK11 (forward 5′-CACCGTGATGGAGTACTGCGTGT-3′; reverse 5′-AAACCACACGCAGTACTCCATCAC-3′).

For lentiviral production, HEK293T cells were seeded in 100 mm dishes at ~1 × 10^6^ cells in 10 mL medium D on day 0. On day 2 (~70%–80% confluence), medium was then removed and replaced with 8 mL medium D supplemented with an additional 2 mM glutamine and the transfection cocktail: 15 μL X-tremeGENE HP DNA Transfection Reagent, 2.5 μg indicated sgRNA-lentiCRISPRv2 construct, 1 μg pMD2.G (Addgene 12259), and 1.5 μg psPAX2 (Addgene 12260). On day 3, medium was removed and treated with 11 mL medium D with 30% FBS supplemented with an additional 2 mM glutamine. On days 4, 5, and 6, virus-containing medium was collected and exchanged with 11 mL of fresh medium D with 30% FBS supplemented with an additional 2 mM glutamine. The collected virus-containing medium was filtered through a 0.45 μm syringe filter unit (MilliporeSigma) and stored at –80°C for future use.

For transduction and selection of monoclonal knockout lines, cells were seeded in 100 mm dishes at ~1 × 10^6^ cell density in 10 mL of medium A (NSCLC line) or medium C (MC38 line). After 24 hours the medium was replaced with fresh medium A (NSCLC line) or medium C (MC38 line) (non-transduced control) or 2.0 mL medium A (NSCLC line) or medium C (MC38 line) and 2.0 mL of the indicated virus-containing medium, supplemented with 8 μg/mL polybrene reagent. After 24 hours, the medium was removed and replaced with 10 mL of medium A (NSCLC line) or medium C (MC38 line). The next day, the medium was removed and replaced with fresh 10 mL medium A (NSCLC line) or medium C (MC38 line) containing 1 μg/mL puromycin, and puromycin-supplemented medium A (NSCLC line) or medium C (MC38 line) was refreshed every 2 days until complete cell death was observed in the non-transduced control. Limiting dilution of these puromycin-selected cells was performed in puromycin-containing medium to produce single colonies of transduced cells, and these monoclonal knockout lines were subsequently propagated and aliquoted for storage under liquid nitrogen or further propagated for experiments described herein.

### CC animal models.

All animal studies were conducted under an IACUC-approved protocol at UT Southwestern Medical Center. NOD/SCID and C57BL/6J mice were obtained from The Jackson Laboratory at approximately 8 weeks of age. All mice were allowed to acclimate in UT Southwestern animal facilities for at least 2 weeks. Animals were kept in a temperature-controlled facility (at approximately 22°C) with a 12-hour light/12-hour dark cycle and were fed regular chow diet.

On day 0 of the study, NOD/SCID and C57BL/6J mice underwent baseline assessments of body weight (digital Ohaus scale) and both lean and fat mass components of body composition using ECHO MRI (ECHO Medical Systems). Then, 150 μL PBS in the absence or presence of either 1 × 10^7^ human NSCLC cells or 1 × 10^6^ human MC38 cells (parental or transduced with CRISPR/Cas9 construct) were injected into the right flank of the NOD/SCID or C57BL/6J mice, respectively. Food intake measurements were conducted daily as described previously, including the method for calculation of average daily food intake (34). Additionally, for Supplemental Table 1 and Figure 1F, a relative average daily food intake was calculated by dividing the average daily food intake of the indicated experimental (cell line–injected) cohort by the average daily food intake of a time-matched control (PBS-injected) cohort. Every 2–3 days, mice underwent longitudinal measurements of body weight, both lean and fat mass components of body composition, and tumor size by caliper (VWR) measurements of length, width, and breadth.

The timing of animal sacrifice for tumor-injected cohorts was a function of imminent animal death (expected within 12 hours), tumor volumes approaching 2.0 cm^3^, or cohorts of animals reaching 45 days of survival; PBS cohorts were sacrificed concurrently with matched tumor cohorts. At sacrifice, mice were euthanized as recommended by the IACUC with use of a CO_2_ chamber. Tumor weights were measured and either immediately processed for FACS analysis (see below) or collected on dry ice prior to long-term storage at –80°C (for all other assays). Whole blood at sacrifice was obtained through cardiac puncture, and serum was obtained by subjecting the whole blood to centrifugation at 960*g* at 4°C for 10 minutes followed by removal of the supernatant; multiplex ELISA evaluation of serum cytokine samples was conducted by Eve Technologies on samples that were 25 μL of mouse serum diluted with 25 μL of PBS.

Longitudinal values for tumor volume were calculated as half the product of caliper measurements of length, width, and breadth. Longitudinal and terminal values for body weight, lean mass, and fat mass were calculated relative to both the matched animal day 0 value and mean value of the matched PBS cohort. At sacrifice, relative tumor-free body weight and lean mass were calculated by subtracting the matched animal tumor weight at sacrifice prior to normalization.

### Gene variant enrichment score.

Whole-exome sequencing and variant discovery for each NSCLC line were available through the COSMIC Cell Line Encyclopedia (v95 2021-Nov-24 release) at https://cancer.sanger.ac.uk (35). To remove synonymous and low-allele-frequency variants from our analysis, we used the variant annotation toolbox *SnpEff*, which maintained only those variants with both (1) DNA allele frequency of 5% or higher and (2) predicted functional impact scores of “MODERATE” or “HIGH,” as defined by ENSEMBL consensus criteria (36). To rank these variants by enrichment in NSCLC cachexia, a GVES was calculated such that 2 points were ascribed if a cell line harbored a high-impact variant in that gene, 1 point if the cell line harbored a medium-impact variant in that gene, or no points for the absence of variant status for that gene. After adding these points up for all cell lines for a given gene, we subtracted the points calculated for the 7 “No CCX” lines from the points calculated for the 10 “CCX” lines to get the final GVES for a given gene. Validation of this scoring methodology for genes was performed by comparing each gene GVES to a continuity-corrected log-odds ratio and using both the GVES and log-odds ratio rank to determine the magnitude of variant association similarly across all 21,200 genes (Spearman’s *ρ* = 0.936, 95% CI 0.934–0.938, *t*_df = 21,198_ = 1,098).

### Clinical patient cohort.

A list of 246 NSCLC patients was procured who had all undergone sequencing and variant detection of serum ctDNA by a Guardant360 CDx assay. The assay identified variants (including single-nucleotide variants, insertions and deletion variants, copy number amplifications, and gene fusions) across 74 genes. All ctDNA analyses for the cohort were conducted within the past 2–3 years, supporting homogeneity in treatment protocols among patients and thus limiting the potential influence of treatment heterogeneity on ctDNA variants identified. Some patients had multiple longitudinal blood collections submitted for ctDNA analysis. As a consequence, we only included the test result at the time of or closest to the patient’s original NSCLC diagnosis. These restrictions led to a final database of 246 patients with ctDNA mutational analysis. For studies on overall survival among other endpoints, we also had long enough follow-up for evaluation of all 246 patients in the cohort. Data on overall survival and other clinical parameters for patients were obtained from the UT Southwestern Harold Simmons Comprehensive Cancer Center Tumor Registry.

This data set was subsequently used to identify associations between tumor gene variants and the presence of cancer-induced weight loss at original cancer diagnosis. The method of identifying patients with cancer-associated weight loss at diagnosis is described forthwith. Briefly, without knowledge of the Guardant data, 1 primary and 1 secondary reviewer separately identified the presence or absence of nonpurposeful weight loss at cancer diagnosis. Patients are routinely weighed as a part of each office visit in our health system, with measurements documented in the electronic medical record. Using these data, we incorporated the validated international consensus definition of CC and pre-cachexia when describing our cohort of patients with cancer-associated weight loss (37). Specifically, we considered cancer-associated weight loss to be present if patients met the criteria of having unintentional weight loss > 5% within 6 months preceding cancer diagnosis if they had a body mass index ≥ 20 kg/m^2^ or unintentional weight loss > 2% with body mass index < 20 kg/m^2^ (both cachexia) or weight loss up to 5% or 2% for the respective body mass indices (pre-cachexia). When multiple measures of weight were available in the pretreatment period, a consistent weight decrease was required for a patient to be classified as having weight loss. Patients with stable weight, weight gain, or purposeful weight loss were classified as not having weight loss. This weight loss was determined numerically if we could identify weights taken prior to a NSCLC diagnosis. Alternatively, a patient was deemed as having wasting if significant (pre-cachexia or cachexia consensus definition level) weight loss was patient reported in a blinded review of symptoms by at least 2 of the following medical professionals — physician (medical oncologist, pulmonologist, radiation oncologist, surgical oncologist, emergency room, etc.) — at the time of diagnosis and/or by at least 1 other medical provider — nurse, nutritionist, dietitian, advanced practice providers, among others. If the 2 reviewers had a discrepancy in their determination of patient weight loss, a third reviewer would adjudicate the outcome.

Association of gene variant status and cachexia at time of cancer diagnosis in this patient population was evaluated for all assayed genes by calculation of the continuity-corrected log-odds ratio, followed by statistical evaluation of the log-odds ratio CI (log-odds ratio *z* test with Bonferroni adjustment for multiple testing). For genes with variants detected in 10 or more patients, the incidence rate of cachexia at time of cancer diagnosis as a function of a specific variant was also calculated and evaluated for statistical significance by contingency analysis (χ^2^ test with Bonferroni adjustment for multiple testing).

### Study approval.

Animal studies were approved through the University of Texas Southwestern Medical Center’s IACUC (protocol 2015-100994). Human serum collected to identify circulating tumor DNA was part of a University of Texas Southwestern (UTSW) Medical Center IRB-approved protocol (STU 2019-1390).

## Author contributions

AYG, JG, TG, JY, PI, and REI designed experiments. PI, JG, TG, JY, AYG, FZ, DW, EML, and JMS conducted experiments and analyzed the data. BG and JDM provided reagents. BME, RH, AK, JDM, and REI analyzed data. CA, VSM, LG, AG, and AYG performed statistical and bioinformatics analysis. AYG, PI, and REI wrote the manuscript. All authors reviewed and revised the manuscript.

## Supplementary Material

Supplemental data

## Version 1 04/24/2023

Electronic publication
