# Supplementary material for: Tumor loss-of-function mutations in STK11/LKB1 induce cachexia
Source: JCI Insight. 2023 Apr 24;8(8):e165419. doi: 10.1172/jci.insight.165419 (PMC10243820; doi:10.1172/jci.insight.165419)
Supplement: Supplemental data [file jciinsight-8-165419-s175.pdf]

SUPPLEMENTAL MATERIALS

SUPPLEMENT MATERIALS AND METHODS

Immunoblot Analysis

Tissue from mouse studies were thawed on ice and approximately 50- 60 mg of tumor tissue was placed in a 2 ml microtubes with 1 ml of Buffer A and homogenized with a Bead Ruptor Elite apparatus (Omni International) for three cycles of 20 s at 4 m/s. The homogenized sample was immediately subjected to centrifugation at 16 °C for 10 min at 10,000 x g. The supernatant was separated from the pellet and collected. Protein concentrations were measured using a Pierce™ bicinchoninic acid kit. Animal tissue and cell culture extracts were mixed with 5X loading buffer, heated at 95 °C for 10 minutes, and subjected to 8% or 11% SDS/PAGE (8 µg/lane). The electrophoresed proteins were transferred to nitrocellulose membranes using the Bio-Rad Trans Blot Turbo system, followed by incubation in blocking buffer. Blocked membranes were washed briefly in PBS-T, followed by primary staining with the following IgG-β-actin (1:1,000 dilution) or IgG-STK11 (1:1,000 dilution). Membranes were again washed in PBS-T, followed by secondary staining with donkey anti-mouse IgG conjugated to horseradish peroxidase. Membranes were again washed in PBS-T, and bound antibodies were visualized by brief incubation in chemiluminescent substrate solution followed by imaging using an Odyssey FC Imager Dual-mode Imaging System (2-min integration time). Immunoblot images were analyzed using Image Studio software (LICOR).

Fluorescence Activated Cell Sorting (FACS) and Flow Cytometry

Approximately 200 mg tumor tissue at sacrifice were collected into ice-cold medium B, and subsequently cut into small pieces and digested with 125 U/mL collagenase type IV and 60 U/mL DNase I type IV for 60 min at 37°C (*1*). The post-digestion samples were passed through a 70-µm cell strainer, the filtrates were pelleted by centrifugation, and the cell pellets were washed with PBS. The washed cell pellets were mixed with anti-mouse CD16/32 monoclonal antibody and incubated on ice for 5 min to block Fc receptors. The blocked samples were then supplemented with antibody cocktail containing fluorochrome-conjugated monoclonal antibodies directed against multiple cell-surface markers and incubated on ice for 30 min in the dark (*2*), after which the cells were washed with staining buffer and

fixed with IC Fixation Buffer. Fixed cells were maintained on ice until analyzed by LSR II or LSRFortessa™ flow cytometers (BD Biosciences) using the gating strategy defined in **Supplemental Figure S1**, and the data from flow cytometers were further analyzed by FlowJo Software (BD Biosciences).

Real-time PCR Analysis of Gene Expression

Extraction of total RNA with RNA-STAT-60 reagent from tumors and subsequent quantitative reverse transcriptase PCR assays for mRNA levels of the indicated gene products were conducted as previously described (*3*). The sequences of primers (obtained from Integrated DNA Technologies) used in these studies are as follows:

Gene Product	Origin	Symbol	Forward	Reverse
β-actin	human	*ACTB*	CACCATTGGCAATGAGCGGTTC	AGGTCTTTGCGGATGTCCACGT
	mouse	*Actb*	CCGTGAAAAGATGACCCAGATC	CACAGCCTGGATGGCTACGT
C-C Motif Chemokine Ligand 5 (CCL5)	human	*CCL5*	CATCCTCATTGCTACTGCCCT	CCTTGACCTGTGGACGACTG
	mouse	*Ccl5*	TTGCAGTCGTGTTTGTCACTC	ATGCTGATTTCTTGGGTTTGCT
Chemokine (C-X-C) motif ligand 12 (CXCL12)	human	*CXCL12*	GCACTTTCACTCTCCGTCAG	TTCAGCCGGGCTACAATCTG
	mouse	*Cxcl12*	CATCAGTGACGGTAAACCAG	GTTTAAAGCTTTCTCCAGGTACTC
Chemokine (C-X-C) motif ligand 2 (CXCL2)	human	*CXCL2*	CCGAAGTCATAGCCACACTC	TCTCTTTCCTCTTCTGTTCCTG
	mouse	*Cxcl2*	CCCAGACAGAAGTCATAGCC	AGTTAGCCTTGCCTTTGTTCAG
Chemokine (C-X-C) motif ligand 3 (CXCL3)	human	*CXCL3*	AAACCGAAGTCATAGCCACAC	TCTCCTGTCAGTTGGTGCTC
	mouse	*Cxcl3*	ACAGAAGTCATAGCCACTCTC	AAGCAGGTAAAGACACATCCA
Chemokine (C-X-C) motif ligand 7 (CXCL7)	human	*CXCL7*	GAGCCTCAGACTTGATACCA	TCTAGACTTTCCTCTTTGCCT
	mouse	*Cxcl7*	GCTCACTGCTCTTCATTATGG	TTCAGTGTGGCTATCACTTCC
Major Histocompatibility Complex, Class II, DR β 1	human	*HLA-DRB1*	CAGAGCAAGATGCTGAGTGG	GAATCCTGTTGGCTGAAGTCC
Interleukin 1β (IL-1β)	human	*IL1b*	CTAAACAGATGAAGTGCTCCTTCC	CACATAAGCCTCGTTATCCCA
	mouse	*Il1b*	TGGACCTTCCAGGATGAGGACA	GTTCATCTCGGAGCCTGTAGTG
Interleukin 6 (IL-6)	human	*IL6*	GAGAAGATTCCAAAGATGTAGCC	GCAAGTCTCCTCATTGAATCC
	mouse	*Il6*	TCGTGGAAATGAGAAAAGAGTTG	AGTGCATCATCGTTGTTCATACA
Leukemia inhibitory factor (LIF)	human	*LIF*	TTATTCTCTATTACACAGCCCAGG	TACACGACTATGCGGTACAG
	mouse	*Lif*	AGCCGTTTCCCAACAACGT	CCGTTGCCATGGAAAGAT
Tumor Necrosis Factor a (TNFα)	human	*TNF*	CTCTTCTGCCTGCTGCACTTTG	ATGGGCTACAGGCTTGTCACTC
	mouse	*Tnf*	CTGAGGTCAATCTGCCCAAGTAC	CTTCACAGAGCAATGACTCCAAAG
Growth Differentiation Factor 15 (GDF15)	human	*GDF15*	CAACCAGAGCTGGGAAGATTCG	CCCGAGAGATACGCAGGTGCA
	mouse	*Gdf15*	CTCTCAACTGAGGTTCCTGC	CCAATCTCACCTCTGGACTG

Microscopic Analysis of Mouse Adipose and Muscle Tissue

Epididymal white fat was collected and immersion fixed in 10% neutral-buffered formalin. All samples were paraffin processed by members of UT Southwestern's Histo Pathology Core using standard histologic technique (4-5). Resulting embeds were sectioned at 5µm, and produced slides were stained by regressive methodology utilizing Leica Selectech reagents (Hematoxylin 560, Define, Blue Buffer, and Eosin-Y 515) on a Tissue-Tek Prisma Plus robotic stainer (Sakura-USA). From the stained slides, microscopy fields were imaged on a BZ-X scanner (Keyence) at 20X magnification and adipocyte area was manually measured using ImageJ software (NIH) for three fields from each slide (250-350

adipocytes per slide). Each group contains the quantification from three slides prepared from three independent mice. Publication proofs of representative fields were obtained on the Keyence BZ-X scanner at 20X magnification.

Whole gastrocnemius skeletal muscle samples were obtained from the limb contralateral to the tumor-implanted flank and cryo-embedded in transverse myofiber orientation protruding from a 1:8 volume mixture of Gum Tragacanth powder (Sigma-Aldrich) to Tissue Freezing Medium (TFM; Triangle Bioscience). All embeds were snap frozen in an isopentane heat extractant bath supercooled to freezing temperature of -155°C in liquid nitrogen. Resulting blocks were stored at -80°C prior to sectioning. Subsequently, gastrocnemius cryoblocks were equilibrated to cutting temperature in a Leica CM3050S cryostat and eight-micron sections were prepared from the transverse face of each block (-25°C object temperature, -23°C chamber temperature, 3.5° blade angle). Slides were briefly air-dried and returned to -80°C for future staining. Slides were thawed from -80°C storage and routine hematoxylin and eosin staining was performed by progressive staining methodology (4-5). In brief, thawed cryosections were first fixed in 10% neutral-buffered formalin for five-minutes and rinsed in running tap water. Fixed cryosections were stained with hematoxylin by five one-second dips in Selectech 560 (Leica Biosystems) and blued in running tap water for five minutes. Once blued, hematoxylin-stained slides were partially dehydrated in 95% ethanol with five one-second dips and then stained with Selectech Alcoholic Eosin-Y 515 (Leica Biosystems) also by five one-second dips. Hematoxylin and eosin-stained slides were immediately destained in three baths of 100% ethanol for five one-second dips each, dehydrated in a fourth 100% ethanol for ten one-second dips, and cleared through a series of three xylene baths for ten one-second dips each. Coverslips were applied with Permount synthetic mounting media (Fisher Scientific). From the stained slides, microscopy fields from the anterior aspect of each gastrocnemius' medial head were imaged at 4X magnification for morphometric analysis. A Leica DM2000 compound microscope equipped with infinity plan-achromat bright-field optics and a Jenoptik ProGres Gryphax NAOS CMOS camera were utilized. Contiguous myofibers (150-250 myofibers per slide) were outlined from each anterior-medial gastrocnemius and their myofiber cross-sectional area manually measured in

ImageJ. Each group contains the quantification from three slides prepared from three independent mice. Publication proofs of representative fields were obtained at higher resolution on an Aperio CS2 scanner (Leica Biosystems) at 20X magnification.

Statistical Analysis.

Details of statistical analysis for each experiment can be found in the respective figure legend. Data is presented as mean ± SEM, dot plots ± SEM, dot plots with bars ± SEM, or histogram. For experimental designs with two conditions, unpaired parametric *t*-tests were conducted to investigate significant differences following tests to verify normality and equal variance of both groups. For experimental designs with greater than two conditions, one- or two-way analysis of variance (ANOVA) tests were conducted to examine if there were significant differences in outcomes among groups. If the ANOVA test showed a significant difference, two sample *t*-tests were conducted to identify which pairs of groups yielded significant differences with Dunnett's multiple comparison post-test. For the case of multiple pairwise comparisons, a Bonferroni correction was used to determine the maximum *p* value required to limit the family wise error rate (α_{FWER}) to 5% or lower as indicated. Overall survival (OS) was estimated using the Kaplan-Meier method from diagnosis to death. Log-rank tests were conducted to determine if there were significant differences in overall survival among groups. All statistical analyses were computed using GraphPad Prism 9 software, except for analysis of patient characteristics and Kaplan-Meier analysis for overall survival for the NSCLC patient data set, which were conducted using SAS software.

Supplemental Materials and Methods References

1. T. Takeshima *et al.*, Key role for neutrophils in radiation-induced antitumor immune responses: Potentiation with G-CSF. *Proceedings of the National Academy of Sciences of the United States of America* **113**, 11300-11305 (2016).
2. F. Zhang, A. Little, H. Zhang, Chronic alcohol consumption inhibits peripheral NK cell development and maturation by decreasing the availability of IL-15. *J Leukoc Biol* **101**, 1015-1027 (2017).
3. T. Guo *et al.*, LIFR-α-dependent adipocyte signaling in obesity limits adipose expansion contributing to fatty liver disease. *iScience* **24**, 102227 (2021).
4. Shehan, Dezna C. and Hrapchak, Barbara B. Theory and Practice of Histotechnology, 2nd edition, 1980. Battelle Press.

5. Carson, Freida L. and Hladik, Christa. Histotechnology: A Self-Instructional Text, 3rd edition, 2009. ASCP Press.

SUPPLEMENTAL FIGURES

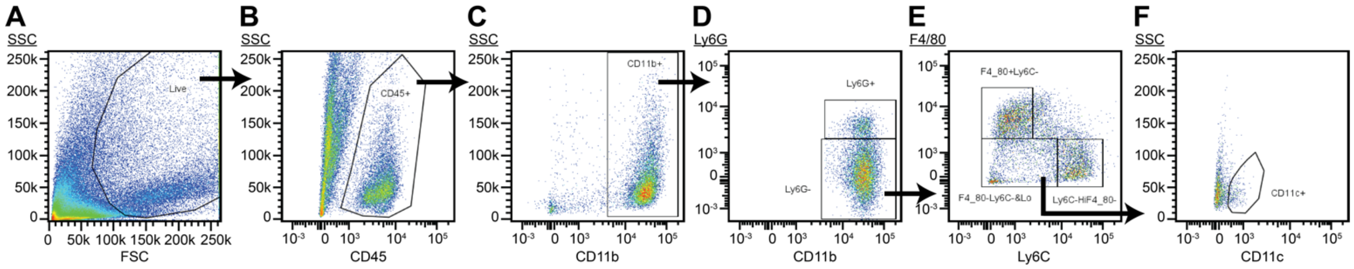

Supplemental Figure 1. Sequential FACS gating strategy for flow cytometry of human NSCLC tumor-infiltrating myeloid cells. A–F) Tumors at sacrifice were processed for FACS/flow cytometry as described in *Methods*. Axes correspond to either forward scatter (FSC) or side scatter (SSC) (linear scale, “K” abbreviates “x 10^3”) or fluorescence intensity of the indicated cell surface protein-directed fluorochrome-conjugated monoclonal antibody (Logicle scaling). FSC and SSC profiles were used to determine live cells (*A*), with the subsequent illustrated gating methods used to determine leukocytes (*B*, $CD45^+$), myeloid cells (C, $CD11b^+$), neutrophils (*D*, $Ly6G^+$), macrophages (*E*, $F4/80^+Ly6C^-$), monocytes (*E*, $F4/80^-Ly6C^{Hi}$), and dendritic cells (*F*, $CD11c^+$).

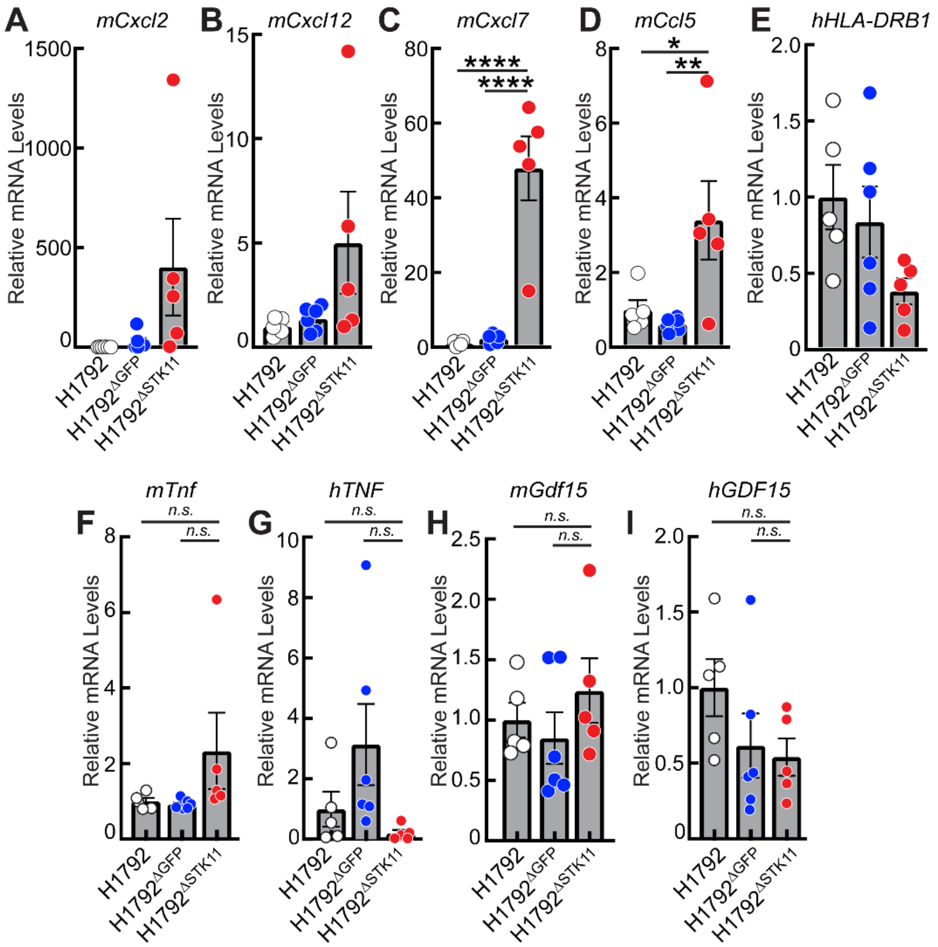

Supplemental Figure 2. Chemokine RNA expression of *STK11*-variant human NSCLCs causing cachexia. (A–I) Tumor samples at sacrifice from the experiment in **Figure 3** were processed for measurement of the levels of tumor mRNA levels relative to H1792 parental cohort of the indicated genes normalized to *β-actin* as described in *Methods*. Data are shown as mean ± SEM. *$p<0.05$, **$p<0.001$, and ****$p<0.0001$ based on one-way ANOVA followed by Dunnett's multiple comparison test for significant differences from the H1792$^{\Delta STK11}$ cohort. *n.s. = non-significant*.

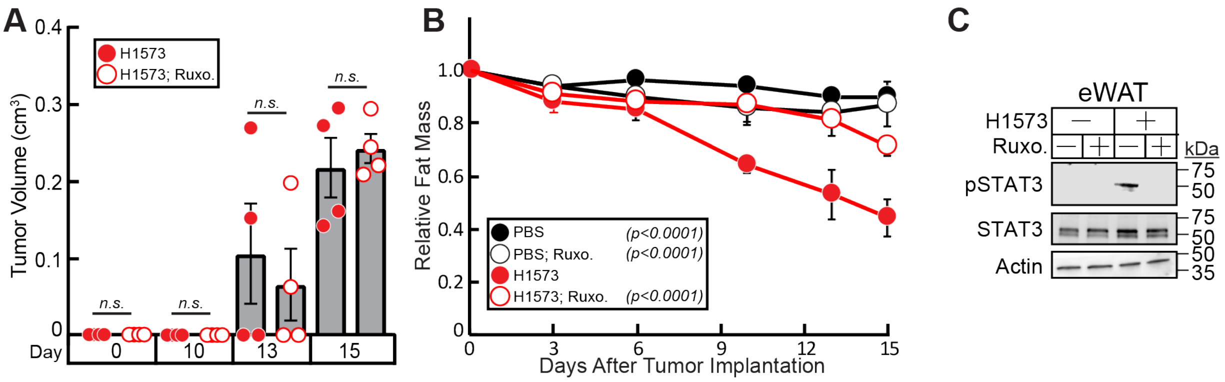

Supplemental Figure 3. JAK inhibition of human NSCLC cachexia-associated adipose wasting. A-C) Chow-fed *NOD/SCID* mice (10-week-old male mice, n = 4 per cohort) were injected s.c. with vehicle or cells from parental H1573 cells followed by i.p. administration of vehicle or ruxolitinib twice daily. Longitudinal measurements of tumor volume (A) and fat mass relative to day 0 values (B) were obtained as described in *Methods*. Epididymal white adipose tissue at sacrifice were processed for immunoblot analysis with the indicated antibodies as described in *Methods (C)*. Data are shown as mean ± SEM. *p* value based on two-way ANOVA followed by Tukey's multiple comparison test for significant differences from the untreated H1573 cohort (A,B). *n.s.* = *non-significant*.

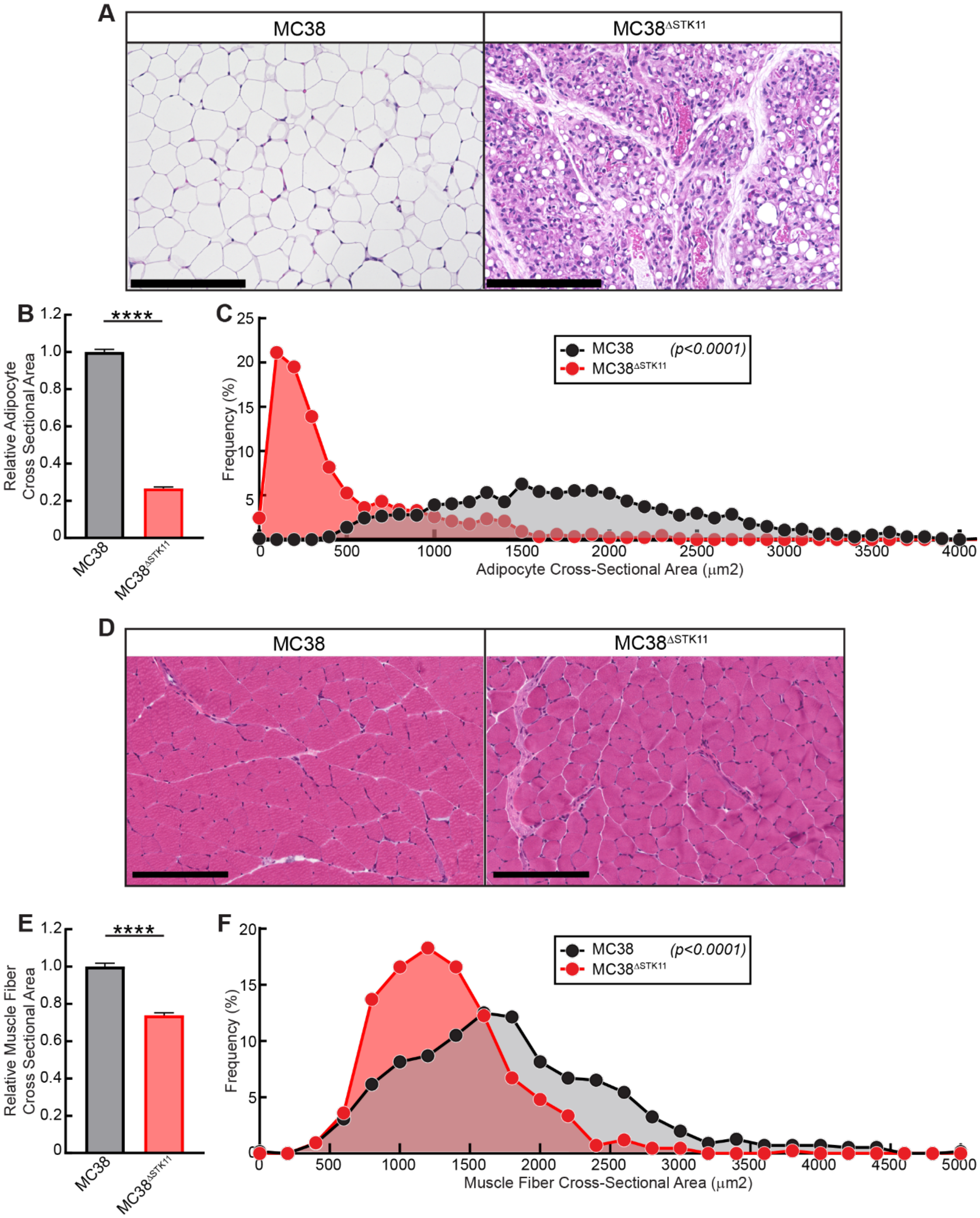

Supplemental Figure 4. Microscopic analysis of adipose and muscle tissues of mice implanted with *STK11/LKB1*-silenced colorectal tumors. A-F) Chow-fed C57BL/6J mice (12 week-old male mice, n = 3 per cohort) were injected s.c. with cells from parental MC38 or MC38$^{\Delta STK11}$ as described in *Methods*. After 21 days, mice were sacrificed and eWAT (A-C) and gastrocnemius muscle (D-F) were harvested and processed for H&E histopathology (A, D; scale bars = 200 μm) with subsequent measurement of adipocyte (B-C) and medial gastrocnemius fiber (E-F) cross-sectional areas. Data are shown as a bar plot with mean ± SEM (B, E) or as a frequency distribution (C, F). ****p<0.0001 or

calculated p-value was determined using an unpaired 2-tailed *t* test for significant differences between the MC38 parental and $MC38^{\Delta STK11}$ cohorts.

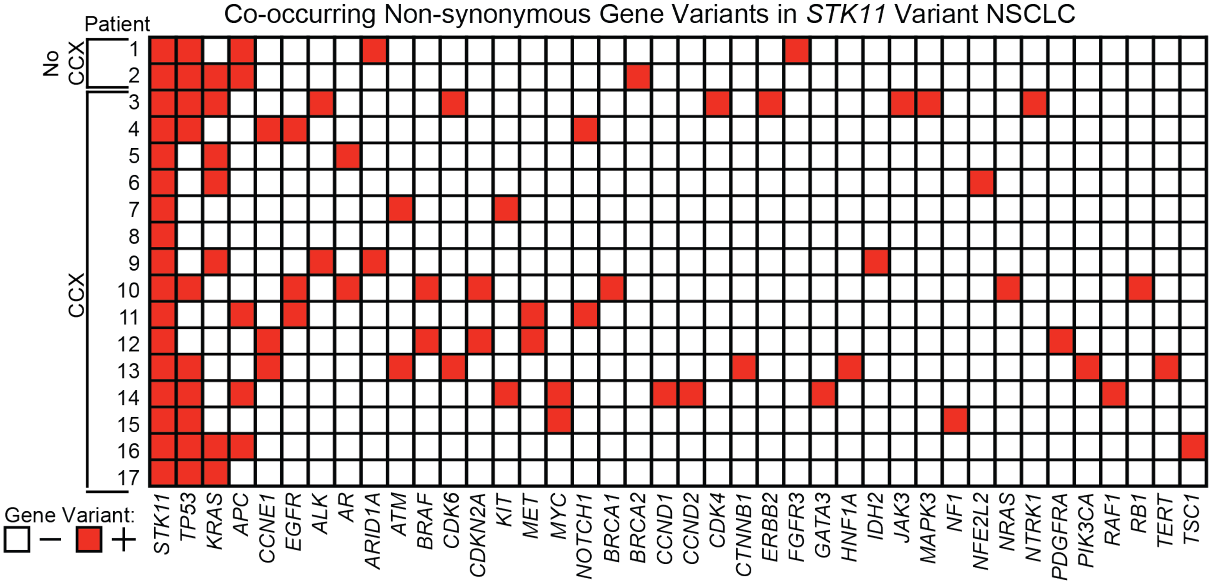

Supplemental Figure 5. Co-occurrence of gene variants in patients with *STK11*-mutated NSCLC
Heat map of other co-occurring gene variants detected in ctDNA from NSCLC patients with *STK11/LKB1*-mutated tumors.

SUPPLEMENTAL TABLES

Supplemental Table 1. Human NSCLC Cachexia Screen: Body Weight, Body Composition, Food Intake and Tumor Growth

hNSCLC Cell Line	Relative Tumor-free Body Weight Loss (%)	Relative Fat Mass Loss (%)	Relative Tumor-free Lean Mass Loss (%)	Relative Daily Food Intake (%)	Terminal Tumor Volume (cm^3)	Days to 0.5 cm^3 Tumor Volume
A427	12.7 ± 1.8	30.5 ± 4.8	10.8 ± 2.4	90.2 ± 10.9	1.8 ± 0.2	34
A549	8.3 ± 1.5	30.6 ± 3.4	7.3 ± 1.9	87.7 ± 8.7	2.3 ± 1.7	14
Calu-3	0.6 ± 2.5	12.0 ± 1.7	-1.2 ± 2.8	100.6 ± 11.8	1.7 ± 0.1	17
Calu-6	14.5 ± 3.1	35.7 ± 5.4	19.6 ± 3.6	98.9 ± 9.6	2.4 ± 0.1	13
DFCI032	4.2 ± 1.0	17.1 ± 6.7	1.1 ± 1.0	108.7 ± 9.4	2.4 ± 0.2	14
H157	5.3 ± 2.2	35.1 ± 14.6	-2.2 ± 3.2	89.9 ± 12.0	2.7 ± 0.5	17
H358	12.2 ± 1.0	16.9 ± 8.5	10.6 ± 0.6	80.3 ± 8.9	2.2 ± 0.2	14
H441	-5.7 ± 1.7	-4.9 ± 11.4	1.1 ± 2.5	84.5 ± 8.6	2.3 ± 0.1	15
H460	6.3 ± 3.9	49.3 ± 6.5	1.2 ± 3.6	87.0 ± 14.9	1.2 ± 0.2	12
H820	3.5 ± 1.5	2.9 ± 4.8	-1.6 ± 2.8	95.7 ± 12.7	1.5 ± 0.4	18
H1155	-2.3 ± 1.7	52.8 ± 4.1	-15.4 ± 2.4	83.0 ± 10.9	1.8 ± 0.3	12
H1299	2.0 ± 2.1	16.7 ± 10.1	-1.4 ± 3.2	88.8 ± 7.1	1.8 ± 0.2	22
H1355	12.6 ± 1.7	31.7 ± 6.3	9.2 ± 1.8	86.2 ± 7.7	0.7 ± 0.3	31
H1373	3.9 ± 0.8	1.4 ± 6.4	2.7 ± 1.2	94.5 ± 10.1	2.0 ± 0.1	14
H1395	11.9 ± 2.2	38.7 ± 4.3	9.5 ± 1.9	93.5 ± 14.7	2.1 ± 0.1	15
H1437	12.6 ± 2.0	53.2 ± 3.7	5.3 ± 2.3	99.1 ± 11.0	2.0 ± 0.2	19
H1568	13.1 ± 1.2	33.7 ± 6.7	8.8 ± 1.9	96.8 ± 15.6	1.5 ± 0.3	28
H1573	18.6 ± 1.5	58.4 ± 3.8	13.9 ± 2.1	103.6 ± 13.8	0.7 ± 0.1	25
H1648	9.8 ± 1.4	41.9 ± 3.5	5.4 ± 1.3	85.5 ± 14.1	2.0 ± 0.2	11
H1650	-0.4 ± 0.7	5.4 ± 6.0	0.8 ± 1.9	99.4 ± 10.0	0.8 ± 0.1	28
H1666	10.7 ± 1.5	35.6 ± 4.4	15.8 ± 2.0	87.0 ± 10.0	2.6 ± 0.3	21
H1703	17.1 ± 2.6	20.5 ± 7.4	17.2 ± 2.3	79.0 ± 10.5	1.9 ± 0.3	20
H1770	2.5 ± 1.6	13.8 ± 5.0	1.2 ± 1.1	100.8 ± 8.9	1.2 ± 0.1	17
H1792	-1.4 ± 0.4	4.3 ± 1.8	-2.1 ± 0.7	100.7 ± 8.0	2.1 ± 0.2	14
H1944	23.2 ± 2.6	50.4 ± 1.3	21.3 ± 3.1	88.4 ± 12.5	2.1 ± 0.5	18
H1975	12.6 ± 1.5	27.5 ± 5.4	7.8 ± 1.5	85.2 ± 9.9	2.4 ± 0.2	8
H1993	18.2 ± 4.2	45.2 ± 4.8	16.4 ± 4.9	80.5 ± 8.1	1.7 ± 0.3	20
H2009	-2.0 ± 1.9	9.4 ± 7.2	-10.5 ± 4.7	111.4 ± 9.8	1.2 ± 0.1	21
H2030	9.0 ± 1.9	26.6 ± 8.8	5.5 ± 1.6	92.9 ± 7.5	1.7 ± 0.3	37
H2087	2.8 ± 0.7	2.2 ± 1.9	1.0 ± 1.8	101.1 ± 8.2	1.3 ± 0.2	40
H2122	10.6 ± 2.9	39.5 ± 7.5	5.7 ± 2.3	95.0 ± 9.1	1.7 ± 0.1	8
H2126	-0.4 ± 1.2	17.2 ± 7.4	-3.7 ± 2.2	102.6 ± 12.6	0.8 ± 0.2	34
H2172	2.2 ± 1.4	13.3 ± 8.1	1.5 ± 1.1	97.3 ± 9.1	1.6 ± 0.1	24
H2887	10.5 ± 0.6	15.1 ± 4.9	11.5 ± 0.7	85.5 ± 10.6	0.8 ± 0.1	27
H3122	8.5 ± 1.4	31.0 ± 11.7	10.5 ± 1.4	99.2 ± 10.0	0.9 ± 0.1	25
H3255	10.7 ± 1.0	15.2 ± 4.5	10.6 ± 1.7	100.1 ± 11.0	0.9 ± 0.1	27
HCC15	3.5 ± 1.8	4.9 ± 7.4	1.1 ± 1.3	92.4 ± 11.1	2.3 ± 0.1	18
HCC44	8.7 ± 1.4	15.0 ± 7.3	8.5 ± 1.2	96.1 ± 7.6	0.7 ± 0.1	43
HCC95	6.0 ± 1.5	8.7 ± 10.2	4.6 ± 2.1	107.4 ± 13.3	1.7 ± 0.3	14
HCC515	15.1 ± 0.5	58.1 ± 2.1	8.9 ± 0.9	80.8 ± 9.8	0.8 ± 0.2	21
HCC827	17.0 ± 2.7	33.4 ± 9.0	12.6 ± 1.7	91.5 ± 12.6	1.9 ± 0.1	22
HCC2279	0.3 ± 1.7	3.4 ± 3.3	-1.2 ± 1.9	96.2 ± 7.4	2.0 ± 0.2	11
HCC4006	9.3 ± 1.0	13.0 ± 6.1	12.7 ± 1.2	95.1 ± 7.4	1.4 ± 0.2	25

Data are shown as mean (Days to 0.5 cm^3 Tumor Volume) or mean ± SEM (all other values) for six mice per cell line cohort, except for cell lines H1299, HC1355, H1944, H2030, H2087, and H2126 in which one mice died before sacrifice.
Values were calculated as described in Methods.

Supplemental Table 2. Patient Demographics and NSCLC Characteristics

Variable	All	No Weight Loss	Weight Loss	*P* Value†
Cachexia status*	246 (100.0)	162 (65.9)	84 (34.1)	
Age - yr‡	65.9 ±11.5	65.0 ±12.1	67.5 ±10.1	0.115
Sex				0.445
Female sex	124 (50.4)	85 (52.5)	39 (46.4)	
Male sex	122 (49.6)	77 (47.5)	45 (53.6)	
Race & Ethnicity‡				0.597
Caucasian, Non-Hispanic	179 (72.8)	117 (72.2)	62 (73.8)	
Hispanic	21 (8.5)	14 (8.6)	7 (8.3)	
Black	34 (13.8)	20 (12.4)	14 (16.7)	
Asian	18 (0.07)	14 (8.6)	4 (4.8)	
Stage‡				0.860
I/II (%)	38 (15.4)	26 (16.0)	12 (14.2)	
III/IV (%)	208 (84.6)	136 (84.0)	72 (85.8)	
Histology‡				0.130
Squamous (%)	34 (13.8)	18 (11.1)	16 (19.0)	
Non-squamous (%)	212 (86.2)	128 (88.9)	60 (81.0)	

Plus-minus values are means ±SD. All other data are show n as number of subjects (% of total).

* Weight loss at cancer diagnosis status w as determined as in *Methods* .

† P values are for the comparison betw een *No Cachexia* and *Cachexia* groups, and w ere calculated w ith the use of either an unpaired parametric t-test (Age at Diagnosis) or a chi-square test (all other variables).

‡ Data for *Age* , *Stage* , and *Histology* correspond to values at time of diagnosis, determined and defined as described in *Methods* .

Supplemental Table 3. Materials for Assays

COMMERCIAL REAGENT	SOURCE	IDENTIFIER
**Antibodies**		
Mouse monoclonal anti-CD16/CD32	BD Biosciences	2.4G2
Rabbit monoclonal anti-STK11	Cell Signaling Technology	3047
Rabbit monoclonal anti-AMPKα	Cell Signaling Technology	2603
Rabbit monoclonal anti-Phospho-AMPKα (T172)	Cell Signaling Technology	50081
Rabbit monoclonal anti-Phospho-Stat3 (T705)	Cell Signaling Technology	9145
Rabbit monoclonal anti-Stat3	Cell Signaling Technology	12640
Mouse monoclonal anti-β-Actin	Cell Signaling Technology	3700
Mouse monoclonal APC-eFluor 780-labeled-anti-Ly6G	Thermo Fischer Scientific (eBioscience)	1A8
Mouse monoclonal APC-labeled-anti-CD11c	Thermo Fischer Scientific (eBioscience)	N418
Mouse monoclonal FITC-labeled-anti-CD45	Thermo Fischer Scientific (eBioscience)	30-F11
Mouse monoclonal PE-Cy7-labeled-anti-CD11b	Thermo Fischer Scientific (eBioscience)	M1/70
Mouse monoclonal PE-labeled-anti-F4/80	Thermo Fischer Scientific (eBioscience)	BM8
Mouse monoclonal PerCP-Cy5.5-labeled-anti-Ly6C	Thermo Fischer Scientific (eBioscience)	HK1.4
Peroxidase AffiniPure Donkey anti-Mouse IgG	Jackson ImmunoResearch	715-035-150
Peroxidase AffiniPure Goat Anti-Rabbit IgG	Jackson ImmunoResearch	111-035-003
**Chemicals and Recombinant Proteins**		
Bovine serum albumin (BSA), molecular biology grade	RPI	A30075
Chloroform	Millipore Sigma (Sigma Aldrich)	372978
Collagenase Type IV	Thermo Fischer Scientific (Gibco)	17104019
DNase I type IV	Millipore Sigma (Sigma Aldrich)	AMPD1
FACS IC Fixation Buffer	Thermo Fischer Scientific (eBioscience)	00-8222-49
FACS Staining Buffer	Thermo Fischer Scientific (eBioscience)	00-4222-26
Non-fat powdered milk	Crystalgen	300-576-010
PBS-TWEEN® Tablets (PBS-T)	Millipore Sigma (Sigma Aldrich)	524653
Phosphatase inhibitor cocktail Set I	Millipore Sigma (EMD Millipore)	524624
Phosphatase inhibitor cocktail Set II	Millipore Sigma (EMD Millipore)	524625
Pierce™ Bicinchoninic Acid Kit	Thermo Fischer Scientific	23225
Precision Plus Protein Kaleidoscope Standards	BioRad, Inc	1610375
Protease inhibitor cocktail	Calbiochem	539131
RNA-STAT-60	Tel-Test	CS-111
SuperSignal™ West Pico PLUS Chemiluminescent Substrate	Thermo Fischer Scientific	34580
**Cell Lines and Tissue Culture Reagents**		
MC38 cell line	Kerafest	ENH204-FP
DMEM with high glucose	Millipore Sigma (Sigma Aldrich)	CD6429
Dulbecco’s Phosphate Buffered Saline (DPBS)	Millipore Sigma (Sigma Aldrich)	D8537
Fetal Bovine Serum (FBS)	Corning	35-015-CV
Penicillin-streptomycin Solution	Thermo Fischer Scientific (Gibco)	15140122
Polybrene infection/transfection reagent (10 mg/ml)	Millipore Sigma (EMD Millpore)	TR-1003-G
RPMI 1640 medium with L-glutamine	Corning	10-40-CV
X-tremeGENE HP DNA Transfection Reagent	Millipore Sigma (Roche)	6366546001
**Mice Strains and Diet**		
16% protein diet (Global), irradiated	Teklad	2916
NOD.Cg-Prkdc<scid>/J (*NOD/SCID*) mouse strain	The Jackson Laboratory	001303
C57BL/6J mouse strain	The Jackson Laboratory	000664
